# FACEts of mechanical regulation in the morphogenesis of craniofacial structures

**DOI:** 10.1038/s41368-020-00110-4

**Published:** 2021-02-05

**Authors:** Wei Du, Arshia Bhojwani, Jimmy K. Hu

**Affiliations:** 1 State Key Laboratory of Oral Diseases & National Clinical Research Center for Oral Diseases & Department of Cariology and Endodontics, West China Hospital of Stomatology, Sichuan University, Chengdu, Sichuan China; 2 School of Dentistry, University of California Los Angeles, Los Angeles, CA USA; 3 Molecular Biology Institute, University of California Los Angeles, Los Angeles, CA USA

**Keywords:** Developmental biology, Cell biology

## Abstract

During embryonic development, organs undergo distinct and programmed morphological changes as they develop into their functional forms. While genetics and biochemical signals are well recognized regulators of morphogenesis, mechanical forces and the physical properties of tissues are now emerging as integral parts of this process as well. These physical factors drive coordinated cell movements and reorganizations, shape and size changes, proliferation and differentiation, as well as gene expression changes, and ultimately sculpt any developing structure by guiding correct cellular architectures and compositions. In this review we focus on several craniofacial structures, including the tooth, the mandible, the palate, and the cranium. We discuss the spatiotemporal regulation of different mechanical cues at both the cellular and tissue scales during craniofacial development and examine how tissue mechanics control various aspects of cell biology and signaling to shape a developing craniofacial organ.

## Introduction

The vertebrate head is an intricate and complex part of the animal body, composed of organs with diverse functions and types. These craniofacial structures, including the cranium, sensory organs, mandible, temporomandibular joint (TMJ), palate, muscles, and teeth, are all constructed in their own unique forms and shapes to facilitate their functions. The complexity of craniofacial skeletal shapes was well appreciated by early naturalists, such as Johann Wolfgang von Goethe (1749–1832), who coined the word “morphologie”.^1^ Goethe’s study on morphological features set the foundation for the work by D’arcy Thompson, which formally recognized the role of physical laws in shaping biological structures, such as the vertebrate skull, during development and across evolution.^2^ Now a century after Thompson’s morphometric study, craniofacial structures with their diverse shapes and architectures once again serve as important models to investigate the developmental processes and cell biological events that propel organ morphological changes. With the advent of novel imaging and biomechanical techniques, we have gained a deeper understanding of how mechanical forces and other physical quantities regulate craniofacial morphogenesis. These studies unveil the interplay between biochemical and mechanical signals during organ formation and provide targetable pathways and guiding principles for developing new regenerative strategies. Here we will first review different types of physical quantities that contribute to tissue development and shape changes, and then focus on the mechanical regulation of selected examples of craniofacial structures.

## Part I. Sources and transduction of mechanical signals during organ morphogenesis

**Fig. 1:**
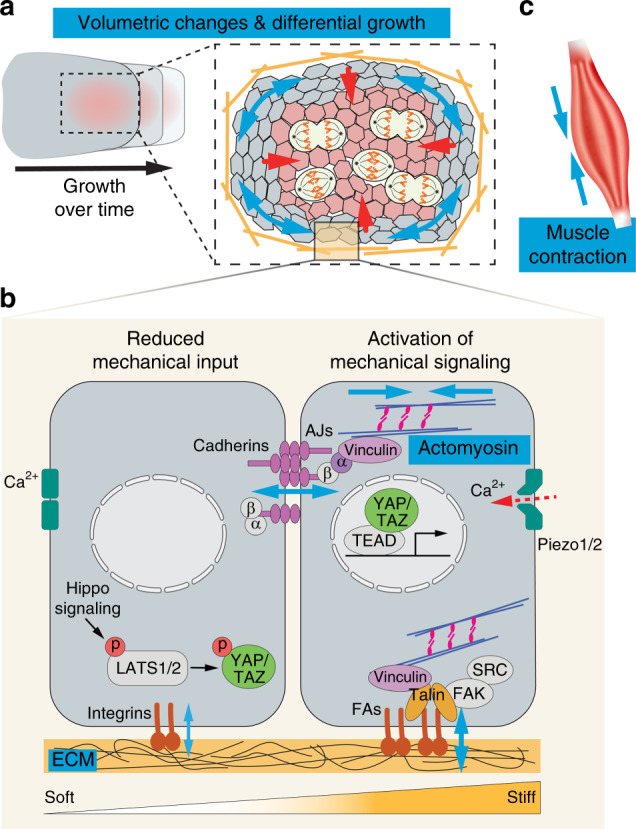
Force generation and signal transduction. Organ morphogenesis is modulated by several different physical quantities: volumetric changes, actomyosin contractility, tissue material property, and muscle contraction. **a** Anisotropic distribution of proliferating cells within a tissue contributes to its directional growth. If the tissue surrounding the proliferating zone does not expand in the same rate, the proliferating zone will experience compression (red arrows); while the surrounding cells will experience tension (blue arrows). **b** Cells generate active forces via actomyosin contractility. Actin cytoskeletons are connected to adherens junctions (AJs) and focal adhesions (FAs), which are mechanosensitive and can mediate increased cell–cell and cell–extracellular matrix (ECM) adhesions, respectively upon increased actomyosin tension and/or substrate stiffness. Both cell adhesion and ECM composition help determine the tissue material properties. The Hippo/YAP/TAZ pathway can also respond to mechanical signals. When there is low mechanical input, the transcription cofactors YAP and TAZ are phosphorylated and restricted in the cytoplasm. When there is high mechanical input, YAP/TAZ are localized to the nucleus and bind to TEAD transcription factors to drive the expression of target genes. Finally, mechanical deformation of cell membranes open up the mechanosensitive Piezo 1 and Piezo 2 ion channels, leading to calcium (Ca^2+^) influx and activation of downstream signaling. **c** Muscle contraction generates large tissue forces that can impact morphogenesis of nearby musculoskeletal elements. Blue arrows represent force directions. α, α-catenin; β, β-catenin; FAK, focal adhesion kinase; p, phosphorylation

Organ morphogenesis is a physical process that integrates mechanical and biochemical information into the regulation of coordinated cell property and behavior changes.^3,4^ There are four main categories of mechanical inputs during development: (1) tissue volumetric changes; (2) generation of cellular forces by cytoskeletons; (3) large scale forces by muscle contraction; and (4) tissue material properties (Fig. 1). Below we discuss the molecular and cellular setup of each process, as well as their functional contribution to organ shape changes. Craniofacial examples are included when applicable. It should also be noted that while these are separate physical quantities, they are often coregulated and interconnected during organ development. Finally, we will discuss the signaling process through the Hippo pathway and Piezo ion channels that convert mechanical inputs into biochemical signals within cells.

### Differential tissue growth and volumetric changes

In a developing organ, progenitor cells can divide, apoptose, and change in size; all of which contribute to the overall growth of the tissue. Spatiotemporal regulation of these processes can therefore lead to differential growth and shape changes. For instance, spatially localized proliferation has been observed at the ventral edge of the developing opercle, a dermal bone of the zebrafish craniofacial skeleton, and this inhomogeneous distribution of cycling cells is responsible for sculpting the correct shape of the bone.^5^ Similarly, during avian beak formation, localized proliferation zones exist in the frontonasal process of different avian species, and spatiotemporal control of the location and size of these proliferation zones directly determines the beak shape in birds.^6^ Differential growth also affects tissue mechanics. As cell numbers and tissue volume increase within a space constrained by surrounding tightly connected cells and/or the extracellular matrix (ECM), the expanding population would experience an increased pressure (compression) and stretch the surrounding cells, which would experience increased strain^7^ (Fig. 1). These force changes have been shown to function as a mechanical feedback to further alter cell behaviors and induce cell differentiation, proliferation, or cell rearrangement.^8–13^

Proliferation can also occur in a directional (anisotropic) manner. As cells often divide along the long axis of tissue elongation, such as during the outgrowth of the vertebrate limb bud and the Drosophila wing disc, oriented cell division has been thought to contribute to tissue lengthening.^14–16^ However, division orientation may be a cellular response to dissipate preexisting anisotropic stresses (forces) within the tissue,^17^ as opposed to driving shape changes directly. Indeed, randomizing cell divisions does not significantly affect morphogenesis, as demonstrated in the developing Drosophila wing disc and during zebrafish gastrulation.^18,19^ Consistent with these findings, proliferation alone cannot account for the morphological changes observed in the developing vertebrate limb bud and mandibular arch,^20,21^ highlighting the importance of other mechanical inputs, such as actomyosin tension and tissue material properties in regulating organ morphogenesis.

### Force generation by cytoskeletons

Actin microfilaments and microtubules are dynamic cytoskeletal structures and components of force generating machineries that convert energy from ATP or GTP hydrolysis into pushing or contractile forces. These forces propel various cellular processes, including cell migration, cell shape changes, and transportation of organelles. Pushing forces are produced when these filaments polymerize against a barrier, such as the cell or nuclear membrane.^22,23^ Contractile forces are primarily produced by the interaction between actin and the motor protein, non-muscle myosin II (MyoII), where activated MyoII assembles into bipolar filaments that crosslink and slide filamentous actin in opposite directions.^24^ Actomyosin tension is critical for many morphogenetic events and spatiotemporal control of the contractile machinery and MyoII activity underlies an important mechanism for generating the anisotropic stress required to deform cells and morph developing tissues.^25,26^ One such example is apical constriction during epithelial invagination. Prior to invagination, signaling cues organize actomyosin cables at the apical side of cells in an epithelial monolayer and apically activate MyoII-dependent contraction via the small GTPase RhoA and Rho-associated coiled-coil kinase (ROCK), effectively shrinking the apical cell surface and driving epithelial buckling.^27,28^ This type of actomyosin-driven shape changes has been implicated in the folding of lens and inner ear placodes, as well as the tongue circumvallate papillae.^29–31^ Polarized Rho and MyoII activity has also been observed in tissues patterned by planar cell polarity, such as during body axis elongation. In this context, Rho kinase and active MyoII concentrate at the cell junctions perpendicular to the elongating axis.^32–34^ The resultant increase in anisotropic actomyosin tension shortens that cell-cell boundary and allows neighboring cells to intercalate along the direction of boundary contraction, thus generating convergent extension movement and tissue elongation.^32–38^

Tissue shape changes, such as those driven by apical constriction and convergent extension, require transmission and coordination of forces produced by individual cells at the supracellular level. In the epithelium, cells are joined to each other via cell adhesion proteins, including the membrane-spanning E-cadherin and P-cadherin in the adherens junctions (AJs). The cytoplasmic tails of cadherins bind to β-catenin, which connects actin filaments to cadherins via α-catenin.^39^ The maturation of AJs and their stable attachment to actin cables are mechanosensitive. Tensional forces transmitted through cadherins and actins unfold α-catenin from an autoinhibited state to an open conformation that allows vinculin binding; vinculin in turn becomes activated to stabilize α-catenin conformation and to promote further actin assembly at AJs.^40–44^ Concurrently, cell contractility can alter cadherin function and junctional integrity.^45–47^ The mechanosensory function of AJs thus allows cells to dynamically react and coordinate mechanical forces at the supracellular level and to adjust adhesion strength for tissue remodeling.

In addition to actin-based cell mechanics, forces originating from non-centrosomal microtubules can also contribute to morphogenetic changes. Microtubules are characterized by their high bending rigidity and capable of bearing compressive stress to maintain cell shapes.^48,49^ During epithelial morphogenesis in Drosophila, cell polarity signals have been shown to reorganize the apical-basal distribution or planar orientation of non-centrosomal microtubules in epithelial cells. This allows tissue level coordination of anisotropic pushing forces generated by microtubule polymerization or dynein-dependent microtubule sliding to modulate cell shapes and overall tissue morphologies.^50,51^ Beyond the direct mechanical control of cells, microtubules can also indirectly influence tissue mechanics by transporting cell adhesion components to targeted regions and promoting local MyoII activation to drive clustering of E-cadherin.^52,53^ Interestingly, mutations in genes encoding factors that are involved in microtubule assembly and dynamics can affect the development of several craniofacial structures in vertebrates, and it will be imperative to determine if microtubule-dependent mechanical regulation plays a role in these processes.^54–57^

### Forces from muscle contraction

Muscles generate forces through the sliding of actin and muscle myosin filaments, and muscle contractile forces have been shown to provide key mechanical signals to regulate the morphogenesis of skeletons, tendons, ligaments, and joints.^58^ Studies using chick embryos with chemically paralyzed muscles and mouse embryos carrying mutations that inhibit muscle formation or contraction have demonstrated that a functional musculature is required for attaining proper bone growth and circumferential shapes of long bones,^59–61^ for promoting the enlargement of bone ridges that attach to tendons,^62–64^ for regulating the size and development of tendons,^65,66^ and for maintaining the fate of joint progenitor cells during joint morphogenesis.^67^ Similar results have also been found in the craniofacial system of several experimental models. For example, mechanical inputs from muscles contribute to the acquisition of species-specific mandible shapes in avian species;^68^ while muscle forces are necessary for the morphogenesis of both the pharyngeal cartilage, as well as cranial tendons in the zebrafish.^69,70^ Consistent with these findings, in both mice and human patients with muscular dystrophy, reduced skull growth and altered craniofacial skeletal shapes are evident, likely as a result of weakened mastication muscles.^71–73^ Muscle contraction is therefore an integral part of morphogenesis and it functions in part by regulating cell rearrangements or ECM remodeling. For instance, muscle forces facilitate skeletal elongation by enabling intercalation of chondrocytes and thus generating cell stacking during bone growth.^69^ The mechanical property of the developing cartilage may also be influenced by muscle contraction, as tensile forces can alter the ECM composition by controlling the expression level of collagens and proteoglycans from chondrocytes.^74,75^ Finally, during tendon development muscle forces can directly affect the ECM organization and stimulate the release of active Tgfβ from the ECM to regulate tendon elongation and branching.^70^ Future studies will focus on how cells sense and convert mechanical signals from muscles to generate specific cell behavior for tissue morphogenesis, which remains an important question in the field.

### Material properties of developing tissues

While tissue growth and cytoskeletons produce forces that enable cell movement during morphogenesis, the extent of ensuing cellular rearrangements and tissue deformations (i.e., the rheological response to forces) depends on the material properties of the developing tissue. These physical properties, such as stiffness and viscoelasticity, are determined by the biochemical and biomechanical states of the constituent cells and their surrounding ECM. Spatiotemporal regulation of tissue material properties can therefore guide morphogenetic events and various cellular processes. For example, a tissue tends to be soft and more fluid-like during early stages of morphogenesis, but cells subsequently increase cortical actin crosslinking and tension that stiffens and maintains the maturing tissue architecture.^76–78^ The viscoelasticity of tissues is further controlled by cadherin-dependent adhesion, as strong cell-cell adhesion can increase the viscosity and the yield stress of the tissue (more solid-like); while reduced adhesion allows tissues to be more fluid-like.^79–81^ In elongating tissues such as during vertebrate body axis extension, establishing a spatial gradient of cadherin-mediated viscoelasticity thus guides progenitor cells through a fluid-to-solid transition, in which cells are initially permissible to rearrange and extend tissues posteriorly in a fluid-like state but become progressively “jammed” anteriorly to preserve the tissue architecture.^81,82^ Such mechanism may similarly function to drive tissue lengthening during craniofacial development, as differential viscosity leading to differences in cell intercalation has been observed during mandibular arch elongation.^21^

ECM is composed of proteoglycans and fibrous proteins (e.g., collagens, fibronectin, laminins), and its composition and structure convey crucial biochemical and mechanical information to guide cell proliferation, differentiation, and movement during development.^83^ Cell-ECM adhesion and signal transduction are primarily through binding of ECM proteins to the transmembrane heterodimeric integrin receptors that are part of the focal adhesions (FAs). In nascent FAs, talin connects cytoplasmic tails of β-integrin subunits to the actin cytoskeleton. Similar to the α-catenin in AJs, the regulation of talin conformation and function is mechanosensitive. In response to an optimal substrate stiffness, cells can exert more forces at FAs via increased actomyosin contractility. Tensile forces at FAs then stretch talin and expose cryptic sites for vinculin binding, which reinforces the talin-actin linkage mechanically and promotes FA maturation.^84,85^ Integrin activation also recruits focal adhesion kinase (FAK) and SRC kinase, which activate downstream biochemical signaling to regulate cytoskeletal organization and gene expression.^83^ FAs therefore allow cells to sense and react to the mechanical property of ECM, and in turn cells can control the matrix stiffness via actomyosin contractility or by modulating the ECM contents and crosslinks.^86^ One example of ECM-guided tissue shape change is the branching morphogenesis of the submandibular gland. Several studies showed that collagens and fibronectin accumulate at the branching point.^87,88^ where integrin activation signals through FAK and RhoA to induce actomyosin contractility.^89,90^ This has two consequences: first it enhances cell motility to facilitate branching; and second, it reciprocally triggers further local fibronectin assembly to support branching structures and promote cell proliferation.^56,89,90^ The ECM can also transduce tissue mechanical forces, for example during the initiation of cephalic neural crest cell migration.^91^ In this context, convergent extension of the head mesoderm results in increased cell density and tissue stiffness. This information is then relayed through the ECM to activate integrin/vinculin signaling in the overlying neural crest cells and induce their migration. The ECM thus plays important roles during craniofacial development. Given that ECM and integrin signaling has been implicated in the morphogenesis of several craniofacial structures that develop from invaginating ectodermal epithelia, including the tooth, optic, otic and olfactory placodes.^92–98^ it is plausible that changes in ECM mechanical properties modulate cell behaviors to facilitate epithelial invagination in these developing organs.

### Sensing and integrating mechanical information with biochemical signaling via the Hippo pathway and Piezo proteins

Above, we discussed the role of integrin signaling in sensing substrate stiffness and then converting that information into biochemical signals to regulate cell behaviors. Another important mechanotransduction pathway downstream of FAs and AJs is the Hippo signaling cascade that controls gene transcription by regulating the activation and nuclear translocation of the transcription cofactors Yes Associated Protein (YAP) and its paralog WW Domain Containing Transcription Regulator 1 (TAZ)^99^ (Fig. 1). The localization of YAP/TAZ in the cytoplasm or nucleus (and thus their transcriptional function) depends on their phosphorylation state. When the Hippo pathway is active, several phosphorylation events lead to the activation of LATS1 and LATS2 kinases, which then phosphorylate YAP/TAZ at several amino acid residues. This restricts YAP/TAZ in the cytoplasm through binding with proteins associated with adhesion complexes, such as 14-3-3 and angiomotin, and promotes YAP/TAZ degradation as well.^100,101^ Conversely, inactivation of Hippo signaling allows unphosphorylated YAP/TAZ to accumulate inside the nucleus and function with other transcription factors to drive the expression of genes that regulate cell proliferation and differentiation. It should be noted that other kinases, including FAK and SRC, can also directly phosphorylate YAP/TAZ,^102,103^ and different signaling mechanisms can be employed to control YAP/TAZ functions. Biomechanically, talin-mediated tension sensing at FAs enables cells to respond to substrate stiffness and trigger actomyosin-dependent YAP activation.^104,105^ Upon integrin engagement, FAK and SRC can additionally signal through PI3K to inhibit LATS1/2 and induce YAP nuclear localization.^106^ Forces transmitted through FAs are also capable of directly deforming the nucleus to allow YAP entry through stretched nuclear pores.^107^ In addition to FAs, AJs are important sites for integrating mechanical signals to the Hippo signaling as well. For example, under low cell density, tension-dependent recruitment of LIM domain proteins Jub (in Drosophila) or LIMD1 and TRIP6 (in mammals) to AJs triggers complex formation of LATS1/2 at AJs, thereby inhibiting LATS1/2 function and promoting YAP activity.^108,109^ By analyzing mouse mutants with organ-specific *Yap* deletion, it was shown that YAP is critically required for the development of several craniofacial structures, including the cranial neural crest, teeth, and palates.^110–112^ However, whether YAP mediates mechanical signals to control aspects of their formation remains to be studied.

Beyond cell junctions, mechanical forces can also be detected by mechanosensitive ion channels, such as Piezo family proteins (Piezo1 and Piezo2), and mutations in *Piezo2* are known to cause several craniofacial syndromes.^113^ Piezo proteins function by responding to pressure and mechanically deformed cell membrane to open its pore for the inflow of positively charged ions, such as Ca^2+^, which in turn activates downstream Ca^2+^-dependent signaling.^114^ Piezo channels thus enable cells in a tissue to sense crowding forces and control cell density through cell extrusion,^115^ and to modulate stem cell proliferation and differentiation in response to tissue mechanical changes.^116,117^ Mechanosensation through Piezo proteins also intersects with Hippo signaling^21^ and how these pathways are coordinated to elicit specific cell responses during development is an intense area of research.

## Part II. Shaping craniofacial structures by forces and material properties

How mechanical forces and tissue properties control organ morphogenesis and cell differentiation is an important developmental question and several craniofacial structures have served as model systems to investigate this subject. These studies have led to paradigms that describe mechanical regulation of various morphogenetic events, as well as integration of biochemical signals that mediate these processes. In this section, we will center our discussion on the mechanical regulation of developing mandibular arches, teeth, palates, jaws, TMJs, and crania, roughly following the order of their developmental initiation. We primarily focus on the mouse as a model organism, where the majority of studies have been conducted.

### Mandibular arch

Pharyngeal arches are transient metameric structures composed of a mesenchymal core and an outer single layer of epithelium that are formed on either side of the developing head at around mouse embryonic day 8–8.5 (E8–8.5).^118,119^ Together with the frontonasal prominence in the medial aspect of the head, these structures undergo extensive outgrowth and morphological alterations to eventually give rise to the face and the neck of an animal. Among them, the first pharyngeal arch (also called the mandibular arch) is subdivided into the dorsally positioned maxillary prominence and the ventrally located mandibular prominence at E9.5. While the maxillary process later forms the upper jaw and the palate, the mandibular process is the precursor of the lower jaw. The epithelium associated with these prominences also gives rise to other craniofacial structures, including the tooth and the salivary gland. Incorrect development of the mandibular arch can therefore cause many craniofacial anomalies with facial and mandibular defects.^120^

**Fig. 2:**
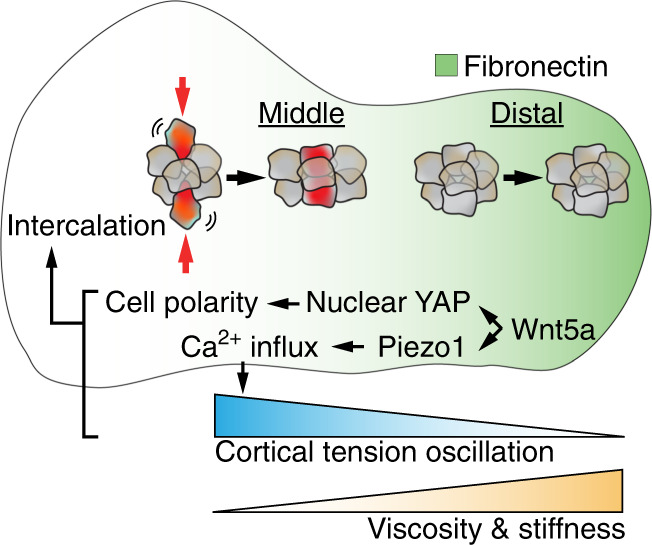
Branchial arch elongation. In the developing mandibular arch, Wnt5a acts upstream of YAP and Piezo1 to control cell polarity and cortical tension oscillations in the middle segment of the arch. This results in increased cell intercalation and tissue fluidity, driving arch elongation. In comparison, the distal arch is stiffer as a result of reduced cell movement and increased deposition of fibronectin

The initial morphogenesis of the mouse mandibular arch involves tissue elongation and bending towards the midline between E8.5 and E9.5. During elongation, the mandibular arch also acquires a morphology characterized by a narrow central waist and a distal bulbous region. While cell proliferation and survival are clearly required for the growth of the arch,^121–123^ how tissue volume changes may drive its lengthening has not been thoroughly studied. However, as the length of cell cycle is similar throughout the middle and distal mandibular process, that quantity alone does not appear to be responsible for the initial morphogenesis of the mandibular prominence.^21^ At the level of signaling regulation, the non-canonical Wnt ligand Wnt5a has been found to be critically required for the outgrowth of epithelium-encapsulated mesenchymal tissues, such as the mandibular arch and the limb bud.^124^ Mutations in *Wnt5a* thus can cause craniofacial abnormalities (and shortened limbs) in both mouse mutants and human Robinow syndrome patients.^125^ Functionally, Wnt5a regulates cell polarity and controls directional cell movement and oriented cell division to propel tissue lengthening.^15,126,127^ In the central segment of the developing mandibular process, Wnt5a acts upstream of YAP/TAZ and the mechano-sensitive Ca^2+^ channel, Piezo1, to induce actomyosin polarity and oscillation of cortical tension as measured using a genetically encoded vinculin tension sensor^21^ (Fig. 2). This reduces local tissue viscosity and facilitates cell intercalation to drive the convergent extension of the arch. The middle arch is therefore more “liquid-like”. In the distal portion of the arch, reduced cell rearrangement stiffens the tissue, which has been demonstrated by measuring the displacement of magnetic beads in the middle and distal regions of the arch using magnetic tweezers.^128^ In addition, distal arch expresses higher amount of fibronectin that also exhibits a mediolateral angular bias. Such spatial variation in ECM abundance and orientation can potentially further contribute to the regulation of arch material property and directional cell movement.^128^ Below we will further examine how different physical properties modulate the development of structures derived from the first branchial arch, including the tooth, the palate, and the mandible.

### Tooth

**Fig. 3:**
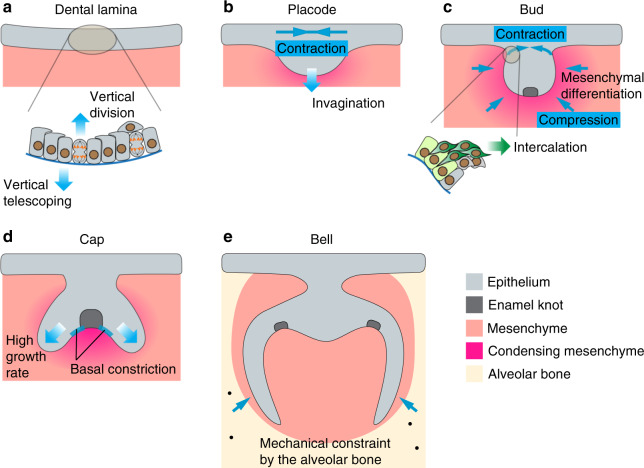
Mechanical regulation of the developing molar. The tooth epithelium undergoes progressive shape changes during its development. **a** At the lamina stage, cells in the epithelial monolayer extend centripetally oriented protrusions to migrate vertically and push neighboring cells towards the mesenchyme (vertical telescoping). Concurrently, vertical cell divisions contribute to epithelial delamination and generation of suprabasal cells. **b**, **c** During the placode and bud stages, suprabasal cells organize their actomyosin cables in the planar orientation and cells (dark green) intercalate towards the center of the bud to generate planar contractile stresses. This mechanically seals the top of the tooth bud and facilitates epithelial invagination by bringing the connecting basal layer cells (light green) towards the center. Concomitantly, mesenchymal cells condense around the dental epithelium and increased compressive stress due to cellular crowding triggers mesenchymal differentiation. **d** The cap shape is postulated to arise as a result of differential tissue growth between the enamel knot (EK) and non-EK epithelium. Basal constriction has also been observed in basal cells neighboring the EK, potentially resulting in the upward buckling of those cells. **e** Mechanical constraints from the alveolar bones play a role in establishing the alignment offsets between the lingual and buccal cusps. Solid blue arrows represent force directions and gradient arrows represent cell or tissue movements

Tooth morphologies are amazingly diverse across different vertebrate species, but their development all begins with the formation of the dental lamina that is discernable as a thickening of the oral epithelium at future tooth sites.^129^ In mice, tooth development begins at around E11, when the dental lamina stratifies and invaginates to form the dental placode.^130^ The stratified dental epithelium then grows further into the underlying cranial neural crest-derived mesenchyme and progresses through increasingly complex morphological changes over time until the tooth erupts. The distinct shapes of the dental epithelium are also used to name each tooth developmental stage: the bud (E12.5–13.5), the cap (E13.5–E15), and the bell (E15-post natal day 7) (Fig. 3). In adults, the tooth crown surface is composed of the enamel, which is generated by dental epithelium-derived ameloblasts during development. Below the enamel is dentin, which is laid down by the neural crest-derived odontoblasts and encloses the dental pulp and the neurovascular bundles within.^131^ Because the tooth is a relatively simple structure during its early development and amenable for ex vivo live imaging, the mouse tooth has become a powerful system to study the cell behavior and associated biomechanical inputs that drive epithelial bending.^132^ This adds to decades of research that have uncovered the reciprocal signaling interactions between the dental epithelium and the mesenchyme,^131^ providing a comprehensive understanding on how mechanical and biochemical signals work in concert to regulate cell movements, divisions, and fate decisions during tooth development.

Similar to other ectodermally-derived organs, dental epithelium begins as an epithelial monolayer, which first bends towards the mesenchyme and then stratifies.^133^ While apical constriction is responsible for the bending of several epithelial organs,^134^ the dental placode clearly utilizes a different mechanism as it lacks apical localization of actomyosin and cells are columnar shaped without apical narrowing.^135^ Instead, dental epithelial cells in mice undergo a process called “vertical telescoping”, where cells send out centripetally-oriented apical protrusions that push on their more centrally located neighbors, and collectively they deform the epithelium downwards.^135^ The formation of these protrusions depends on actin polymerization and branching and requires both hedgehog (Hh) and fibroblast growth factor (FGF) signaling, as chemically inhibiting any of these processes reduces protrusion numbers and abolishes epithelial invagination. The same mechanism also enables the invagination of the salivary gland epithelial monolayer.^135^ In the developing molar, epithelial invagination is accompanied by vertical cell divisions to produce suprabasal cells and FGF signaling functions as a necessary cue to induce cell proliferation and epithelial stratification.^136^ The increase in tissue volume in the suprabasal space can therefore in theory generate pressure to further bend the epithelium. However, to direct this pressure into driving invagination only, a physical barrier needs to be established apically to restrict tissue buckling upwards. This was in part achieved by planarly-oriented tissue tension in the suprabasal cells, which display prominent actomyosin bundles at the supracellular level in the same direction as the tension.^133^ The evidence of tissue tension was demonstrated through a series of mechanical cutting experiments, in which an initially tensed tissue would recoil from the point of cutting. For example, following an incision made in the middle of the mouse molar suprabasal layer, the bisected tissues recoiled in opposite directions and the degree of epithelial bending was reduced, indicating the presence of contractile forces that facilitate invagination. Complementing this finding, an incision made outside the tooth epithelium incurred additional epithelial bending, as the suprabasal contraction was no longer resisted. On the contrary, if an incision was made first in the suprabasal layer to relieve local tension and followed by a lateral cut outside the tooth germ, no recoil was observed. Similarly, if a lateral cut was made on tissues cultured in the presence of Blebbistatin that inhibits MyoII function, no recoil was detected, either. Together these results concretely show that actomyosin-dependent epithelial contraction is integral to the tooth invagination process.

As the molar placode enlarges in volume and gradually transforms into a bud shape, portions of the suprabasal layer continue to narrow and forms a neck region that connects the bud to the surface epithelium. Live imaging of the mouse molar bud showed that this results from a convergent extension type of cellular movement.^133^ In this context, suprabasal cells migrate towards the center of the placode and intercalate with one another. At the same time, basal cells at the edge of the placode are both anchored to their neighbors and attached to adjacent suprabasal cells via E-cadherin, drawing themselves towards the placode center. Collectively, these movements generate even more planar tissue contraction that not only seals the top of the dental placode but also pulls the basal cells in the neck region towards each other in a pinching fashion, effectively driving epithelial buckling toward the mesenchyme.

At E12.5 the developing mouse tooth bud concomitantly induces the underlying mesenchyme to condense. Mesenchymal cells migrate towards the invaginating epithelium in response to the long-range chemo-attractant FGF8 secreted from the tooth bud, which also produces SEMA3F, a short-range repulsive signal, to augment mesenchymal compaction by the epithelium.^137^ The compressive stress from cellular crowding is thought to function as a mechanical signal to initiate mesenchymal cell differentiation, as compressing dissected mandibular mesenchyme in culture promotes expression of odontogenic markers *Pax9* and *Msx1*. Cell crowding during condensation also modulates the ECM composition by inducing the expression of collagen VI.^93^ The presence of a structurally organized ECM is clearly important for maintaining mesenchymal differentiation, as chemical inhibition of lysyl oxidase, which catalyzes collagen crosslinking and therefore regulates ECM stiffness, results in diminished mesenchymal condensation, as well as *Pax9* expression.^93^ Consequently, both condensation-induced compression and changes in the ECM property contribute to the regulation of odontogenesis. It remains unclear whether mesenchymal condensation and the associated material properties also provide mechanical cues to control the development of the overlying epithelium.

The epithelial bud-to-cap transition between E13 and E13.5 in mice coincides with the formation of the tooth signaling center, known as the primary enamel knot (EK).^138,139^ The EK is composed of a group of postmitotic cells that are specialized in signal secretion, expressing various ligands, including sonic hedgehog (SHH), bone morphogenetic proteins (BMPs), and FGFs, which maintain the proliferation and continuous extension of the epithelium surrounding the EK (called the cervical loop).^140^ The cap shape was posited to arise as a result of differential proliferation between the non-dividing EK and the proliferative neighboring cervical loops.^141^ Support for this idea came from tracking the development of cultured molar slices, which showed higher growth rates in the epithelium adjacent to the EK than in the EK itself.^142^ Computational modeling that combines these experimental data with consideration of the physical constraint provided by the less proliferative mesenchyme, and differential adhesion between the mesenchyme and the epithelium, predicts that the tooth bud is guided by these factors to buckle at the presumptive cervical loop areas and to grow downward from those sites.^142–144^ Surprisingly, chemical inhibition of cell proliferation in slice culture does not prevent bud-to-cap morphogenesis,^145^ suggesting that while differential proliferation may contribute to the cap shape, it is in fact not required for this process. Proliferation-independent mechanisms must then exist to initiate the bud-to-cap shape changes. One possible mechanism is through basal constriction of cells on either side of the EK.^145^ Prior to the cap stage, myosin heavy chain IIB and actin bundles were observed to accumulate on the basal surface of cells that are adjacent to the forming EK. Quantifying the shape of these cells at different developmental timepoints between E13.5 and E15.5 in mice further revealed that they have decreased basal width over time.^145^ As a result, it is conceivable that actomyosin tension contracts the basal surfaces surrounding the EK and drives evagination of the inner dental epithelium away from the mesenchyme, thus creating the cap shape. However, this would require further experimental confirmation. The same study also showed that inhibition of FAK signaling abolishes bud-to-cap transition^145^ and therefore perhaps regional activation of integrin/FAK signaling through interactions between the epithelium and the mesenchyme is crucial for shaping the epithelium at this stage. At the same time, given that EK formation depends on α-catenin-mediated inhibition of YAP activity,^146^ it will be interesting to explore how tissue forces generated by cell shape changes, such as the basal contraction described above, regulate YAP localization and activity to control EK differentiation.

Between the cap and bell stages, the cervical loops invaginate further into the mesenchyme and this may be mechanically powered by both increased actin-dependent cell motility and oriented cell divisions along the axis of the extending epithelium.^142^ During the bell stage of mouse molar development between E15.5 and E16.5, the primary EK undergoes apoptosis and secondary EKs are formed.^142,147–149^ Secondary EKs play an important role in determining the cusp locations and crown morphology in multicuspid teeth.^150^ In monocuspid teeth, such as the incisors, only the primary EK is formed. Mechanical constraints from the alveolar bones that surround the developing molars appear to play a role in establishing the amount of offsets (or alignment) between the lingual and buccal cusps.^151^ This was realized because mouse and vole molars cultured as ex vivo explants without the surrounding bones lose their offset patterns but can be rescued by lateral compression imposed by artificial mechanical constraints. Soft tissue tomography also showed that the morphology and growth of molars are strongly associated with those of alveolar bones, highlighting co-development of these tissues and possible mechanical interdependence due to their close proximity. In diphyodont animals (animals that initially have a set of deciduous teeth that are later replaced by the permanent set of teeth), such as the miniature pig, compressive stress due to alveolar constraint has also been implicated in timing the activation of permanent tooth development from an arrested state.^152^ The compressive stress is generated as a result of deciduous teeth growing faster than the expansion of alveolar sockets, and acts as a mechanical signal to induce an integrin β1‐ERK1‐RUNX2 signaling axis in the adjacent mesenchyme, which in turn suspends the permanent tooth epithelium in an arrested state. Once the compression is released after tooth eruption, integrin β1‐ERK1‐RUNX2 signaling is reduced and the permanent tooth proceeds to develop.

Together these studies accentuate the importance of tissue forces during tooth morphogenesis and point to the necessity to consider these mechanical factors when bioengineering human teeth based on developmental principles. For example, designing a hydrogel that matches the elastic modulus of dental tissues supports the formation of biomimetic tooth buds from primary porcine dental cells.^153^ As we learn more about how mechanical signals guide tooth development, increasingly sophisticated mechanical manipulations can be implemented in novel bioengineering platforms through the application of photochemistry and optogenetics that facilitate spatiotemporal control of the hydrogel properties^154^ and cellular forces.^155^ By recreating the mechanical microenvironment and the biochemical-mechanical signaling interactions observed in developing teeth, we will be able to more precisely direct dental progenitor cell proliferation and differentiation in culture and to bioengineer teeth with the correct shape and architecture. Finally, as the dental mesenchyme clearly responds to mechanical cues,^137,156,157^ an in-depth understanding of the mechanical modifiers that influence their fate decision is essential to fully realize their potential for stem cell-based therapies and tissue regeneration.

### Palate

The palate forms the roof of the mammalian mouth and physically separates the oral cavity from the nasal cavity. Anatomically, the palate is consisted of the primary and secondary palates; the primary palate encompasses the triangular region between the incisive foramen and the alveolar ridge surrounding upper incisors, and the secondary palate comprises the rest of the hard and soft palate posteriorly. The primary and secondary palates have distinct embryological origins. Whereas the primary palate is derived from the frontonasal prominence at the rostral anterior side of the mouth, the secondary palates develop as outgrowths from the oral surface of the paired maxillary processes on either side of the mouth. These outgrowths are largely composed of cranial neural crest-derived mesenchyme and surrounded by a layer of oral epithelium.^158^ In mice, the secondary palatal outgrowths become visible at around E11.5, marking the beginning of palatogenesis. Between E11.5 and E13.5, the secondary palatal shelves grow in size and first extend vertically towards the mandible on either side of the tongue, while displaying stereotyped morphologies that are distinct along the anterior-posterior axis. From E13.5 to 14.5, the developing secondary palates undergo palatal shelf elevation and reorient themselves from the vertical orientation to the horizontal position that is above the tongue. The two palatal shelves subsequently grow towards each other and make contact at the midline. The juxtaposed epithelial linings then merge to form the midline epithelial seam (MES), which marks the beginning of palatal shelf fusion at around E14.75. The MES gradually disintegrates and the palatal shelf mesenchyme becomes one confluent structure. At the same time, the secondary palate also fuses with the primary palate anteriorly and with the nasal septum anterodorsally, to form a complete palate by E17.^159^ As a result, palate development involves a series of coordinated tissue movement and remodeling that culminates in the joining of initially separated tissues. Disruptions in this process due to gene mutations or other environmental factors can therefore cause cleft palate, which is one of the most common craniofacial birth defects in human.^160^ For example, mutations in *Tgfb3* or *Irf6* affect proper dissolution of MES and epithelial adhesion, resulting in failed palate fusion in both humans and mice.^161–165^ In fact, mutations in *Irf6* are the most common cause of human cleft lip and/or palate.^166^

**Fig. 4:**
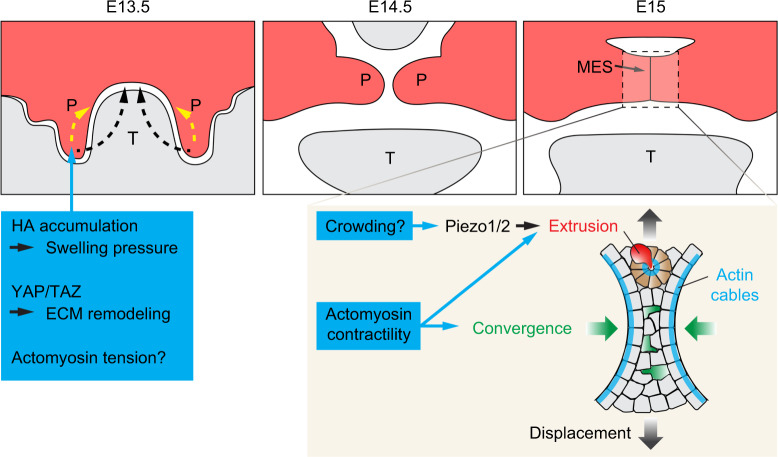
Mechanical regulation of palatal shelf elevation and fusion. Palatal shelves elevate either through a swinging motion (black arrows) or cell reorganizations (yellow arrows). Increased hydration and ECM expansion due to hyaluronic acid (HA) accumulation in the mesenchyme, ECM remodeling, and actomyosin tension have all been postulated to provide the elevating forces. Palatal fusion takes place at the midline epithelial seam (MES). Actomyosin tension is required to promote epithelial cell convergence (green cells and arrows) towards the midline and the subsequent cell displacement (black gradient arrows) towards the periphery. Actomyosin contractility and signaling through Piezo1/2 facilitate the formation of cellular rosettes (brown cells) and cell extrusion (red cell), leading to the removal of MES cells. Lastly, the actin cables at the edge of the MES contract and contribute to MES breakage. P, palatal shelf; T, tongue

Multiple aspects of palatogenesis are thought to require coordinated generation of cell and tissue level forces to direct their movements (Fig. 4). During shelf elevation, the anterior palatal shelves undergo a rapid upward swinging motion to bring the palates from their vertical position to the horizontal position; while the medial and posterior portions of the palatal shelves achieve elevation by controlling the flow and organization of cells to alter the tissue shape.^167,168^ While the exact mechanism remains unresolved, multiple physical properties, such as alterations in the mesenchymal cell density,^169^ regional changes in proliferation,^170^ and remodeling of the ECM and cytoskeletons,^171,172^ have been hypothesized to generate the elevating forces. For instance, in *Osr2* null mouse embryos in which shelf elevation is delayed, proliferation is specifically reduced in the medial half of the downward-pointing palatal outgrowths.^170^Similar phenotypes have also been observed in mutant embryos with conditional *Fgfr1* deletion in the cranial neural crest lineage.^173^ Reduced proliferation can in theory impair the horizontal expansion of the palatal shelves and affect the anisotropic pressure buildup that drives shape changes. Another possible contributor to shelf elevation is ECM remodeling. One of the main components of the palatal shelf ECM is the glycosaminoglycan hyaluronic acid (HA), which accounts for about 60% of the ECM mass.^174^ Prior to shelf elevation, HA accumulates in the palatal mesenchyme and it has been postulated that hydration of HA expands the ECM volume and provides the pressure necessary to elevate the palatal shelf.^175,176^ This idea was recently queried by experiments inhibiting HA synthesis specifically in the shelf mesenchyme via Osr2-Cre-mediated conditional deletion of *Has2* (encoding hyaluronic acid synthase 2).^177^ In these mutants, palatal shelves are reduced in size and undergo delayed, but complete, elevation. This result thus shows that while HA accumulation is intrinsically required for the expansion of the palatal shelf prior to shelf elevation, it is not the only source for generating the elevating force. Interestingly, embryos with *Has2* deletion in both the shelf and mandibular mesenchyme, or just in the mandibular mesenchyme, exhibit mandibular hypoplasia, as well as failed shelf elevation, which can be rescued by culturing the mutant maxilla without the mandible and tongue.^177,178^ Therefore, the mandible and the tongue also require HA for their correct morphogenesis, which is permissive for proper shelf elevation. When malformed, these structures remain as physical obstructions and secondarily block the elevating shelf. Conversely, forces generated by HA hydration within the palatal tissue may help overcome the initial blockade by the tongue during normal palatogenesis, allowing the palate to displace the tongue dorsally in a timely manner; although this force is not required for the eventual shelf elevation.^178,179^

Collagen organization also appears to be important for palatal shelf elevation, as deletion of the collagen crosslinker, lysyl oxidase-like 3 (LOXL3) results in failed elevation.^180^ Similarly, in mouse embryo mutants lacking the transcription cofactors YAP and TAZ in the palatal mesenchyme, palatal elevation is delayed and the expression of *Loxl4* that encodes another lysyl-oxidase family protein, LOXL4, as well as the expression of collagen proteins, are both reduced.^112^ These results thus highlight the importance of ECM remodeling during palatogenesis, although its mechanistic regulation remains an important open question. In addition, given the role of YAP/TAZ in mechanotransduction,^104^ it is plausible that YAP/TAZ may be part of the mechanical feedback loop that both senses and modulates the mechanical property of the developing palate. It should be noted that cartilage-specific conditional deletion of *Yap*/*Taz* using Col2a1-Cre also results in cleft palate in mice, possibly due to malformed Meckel’s cartilage that prevents proper tongue descent.^181^ However, as Col2a1-Cre activity is in fact detectable in a subset of the posterior palate mesenchyme at E12.5 and mutant palatal shelves fail to elevate and fuse in cultured explants of whole maxillae without mandibles and tongues,^112^ the function of the mandible and the tongue to physically block shelf elevation in this context needs to be further examined. Finally, besides HA and collagens, several other ECM molecules have also been found to be expressed in the developing palatal tissue. For example, Tenascin-C and Tenascin-W are predominantly expressed in the medial portion of the shelf mesenchyme prior to its elevation, potentially contributing to differential mechanical properties along the mediolateral axis of the tissue.^171^ The tenascin meshwork also aligns with actin bundles and the long axis of nuclei, which are oriented toward the nasomedial wall of the elevating middle and posterior palatal shelves. These observations thus suggest that actomyosin contractility and tissue material property may play an important role in shaping the middle and posterior palatal shelf during elevation. Future studies determining the functional requirement of actomyosin-based cellular forces in shelf reorientation and the role of tissue material properties in modulating this process will further our understanding of this decades-old question of how palatal shelf elevates.

While the ECM plays an important role during shelf elevation, apoptosis and actomyosin-driven cellular extrusion are integral to the fusion of palatal shelves. Over the past decades, we have gained significant insights into the cellular processes facilitating palatal fusion. Three possible mechanisms have been proposed to drive MES dissolution: (1) epithelial-mesenchymal transition (EMT),^182–185^ (2) apoptotic cell death,^186–188^ and (3) cell migration..^189,190^ Among them, apoptosis is perhaps one of the most researched mechanisms for MES removal. When palatal shelves come in contact at the midline, apoptosis is triggered in the MES, and signaling through retinoic acid and Tgfβ3, as well as Irf6 function, have been shown to be critical for inducing apoptosis in MES cells and palatal fusion.^187,191–194^ Consistent with these results, 45% of the *Bok*^*–/–*^;*Bax*^*–/–*^*;Bak*^*–/–*^ triple knockout mice, where intrinsic apoptosis is blocked, exhibited complete cleft palate.^195^ However, fusion at MES was not specifically examined in these mutants, leaving questions on whether the palate phenotype is caused by defects in other steps of palatogenesis or by non-tissue-autonomous effects.

How then is regulation of apoptosis integrated with cellular processes that drive the merging of epithelial cells during MES formation? This is in part achieved by actomyosin tension-driven cellular convergence and extrusion. Live imaging of mouse mutants with conditional deletion of non-muscle myosin heavy chains IIA and IIB in the palate epithelium showed that actomyosin contractility is required for cell intercalations towards the midline, thus displacing cells from the center of the initially multi-layered MES towards the oral surface.^196^ Similarly, drug inhibition of MyoII upstream regulators, ROCK and myosin light chain kinase (MLCK), in explant culture also blocks cell interaction and palatal fusion.^196^ Therefore, actomyosin tension permits coordinated cellular rearrangement to promote the thinning of the epithelium. Concurrently, as more cells are displaced towards the periphery of MES, they would experience increased crowding and are actively extruded from the epithelium. In this context, actomyosin-dependent formation of cellular rosettes facilitates extrusion of both apoptotic and live cells, and the mechanosensitive Piezo ion channels have been found to promote this process, possibly in response to increased cellular crowding.^196^ Cellular forces generated by actomyosin contraction is therefore critical for palatal fusion at multiple levels.

Later, following secondary palate fusion, the pressure generated by infant suckling, a mammalian-specific feeding behavior, has also been linked to the formation of a temporary cartilaginous growth plate-like structure in the mid-palatal suture that otherwise ossifies primarily through intramembranous ossification.^197^ Using finite element modeling, the computed patterns of suckling-generated distortional and hydrostatic strains in palates correlate with patterns of chondrogenic gene expression. In addition, different parts of the palate structure exhibit distinct mechanical properties,^197^ consistent with the spatiotemporal regulation of various ECM proteins during palatogenesis.^172^ Together, studies discussed here demonstrate that forces of different types and at various scales regulate multiple aspects of palate development. Future research combining genomic, biochemical and biomechanical approaches will help advance our understanding of the mechanical control of palatogenesis, as well as the genetic and cellular responses to physical cues. These efforts will in turn inform targetable mechanical pathways that drive normal palate elevation and fusion, and guide us towards therapeutic intervention to prevent cleft palate birth defects.

### Jaw and temporomandibular joint (TMJ)

The development of the lower jaw first becomes apparent when cranial neural crest-derived mesenchymal cells differentiate into chondrocytes and form a rod-shaped cartilage, known as Meckel’s cartilage, at around E12.5 in mice.^198^ Meckel’s cartilage then extends in length at both ends of the cartilage. At the same time, mesenchymal cells neighboring Meckel’s cartilage begin to condense and differentiate into osteoblasts, which undergo intramembranous ossification to form a set of bony tissues that subsequently fold over the gradually degenerating Meckel’s cartilage. Functionally, Meckel’s cartilage does not appear to be required for the initial formation of the mandible, as the mandibular ossification still takes place in the absence of Meckel’s cartilage, as in *Sox9* null embryos.^199^ However, *Sox9* mutant mandibles are smaller in size, suggesting that Meckel’s cartilage may control the size and shape of the mandible as it develops.^200^ Consistent with this, reduced mechanical integrity in the deformed Meckel’s cartilage of *Ctgf* null mice leads to shortened mandibles.^199^

At the proximal end of the mandible, the TMJ links the jawbone to the temporal bone of the skull, and enables mandibular movement and mastication. The TMJ includes the condylar head of the mandible and the mandibular fossa of the temporal bone; both of which arise from endochondral ossification. A fibrous articular disc further divides the TMJ into two compartments, separating the condyle and the fossa. TMJ development begins at E13.5 when mesenchymal cells condense to form the condylar and temporal blastema, which then grow towards each other while the disc forms in between as a separate condensation at E16.5. The secondary cartilage of the condyle also joins the developing mandible and produces new bones that sustain the continued growth of the mandible.

Like many bones in the vertebrate body, jawbone and TMJ morphogenesis is closely linked to muscle functions. It is therefore not surprising that mechanical forces are important modifiers of mandible development and morphologies both in embryos and postnatally, thus in accordance with the Wolff’s law,^201^ which stated that bone shapes and structures depend on the functional forces of the muscles. In mouse embryos, jaw movement begins at E14 and restricting jaw motility by *exo utero* suturing of the jaw at E15.5 results in a smaller articular disc and a shorter but thicker mandible at E18.5 as a result of reduced chondroprogenitor proliferation and abnormal chondrocyte differentiation in the TMJ and condyle cartilage.^202,203^ During bone development, feedback between Indian hedgehog (Ihh) and Parathyroid hormone-related peptide (PTHrP) is central to the regulation of chondrocyte proliferation and their expression is downregulated in the condyle cartilage of sutured mandibles.^204^ Interestingly, *Ihh* expression can be induced by cyclic stress in cultured chondrocytes,^205^ suggesting that the regulation of *Ihh* transcription is mechanosensitive and can respond to mechanical stimuli to tune bone growth during mandible development. In zebrafish, muscle functions have also been linked to jaw joint development as immobilizing muscles through anesthetization causes jaw joint dysmorphology, particularly in regions of high compressive strain.^206,207^ In this context, Wnt signaling is activated by mechanical stress and biochemically transduces mechanical signals to regulate chondrocyte proliferation, migration, intercalation and cell morphology to shape the Meckel’s cartilage and jaw joint.^208^

Muscle forces continue to shape the mandible in postnatal animals. For example, muscle sizes and bite forces are associated with mandibular shape variations in humans,^209^ and patients with reduced muscle function develop altered craniofacial morphology.^210^ Similarly, decreasing masticatory load in mice by feeding them with a soft diet results in transgenerational inheritance of mandibular shape changes, although the exact mechanism is not understood.^211^Consistent with these observations, altering mechanical forces placed on mandibles and condylar cartilage by feeding animals a soft diet, trimming their teeth, or forced mouth opening, affects chondrocyte biology in several different animal models.^212–218^ These studies showed that mechanical stress is required to promote chondrocyte proliferation, maintain adequate differentiation, and support ECM production. Akin to the developing mandible, *Ihh* expression is also responsive to mechanical loading in the adult condylar cartilage,^219^ pointing to a common mechanism that enables continued adaptive changes in mandibular growth to altering mechanical environments. Importantly, as the primary cilia have been shown to be required for Ihh signaling activation in response to hydrostatic compression in cultured primary epiphyseal chondrocytes^220^ and primary cilia are essential for correct TMJ development,^221^ it will be interesting in the future to assess if primary cilia can mediate mechanical signals to control chondrocyte proliferation in the mandible.

Differences in the mechanical load as a result of differential muscle patterning also have evolutionary consequences. For instance, when compared with quail and chick embryos, the relatively larger mandibular adductor muscles in duck embryos generate a species-specific mechanical environment that signals through FGF and TGFβ signaling to induce the formation of a duck-specific coronoid process for the adductor insertion on the mandibular bone.^68,222^ Another example is the loss of TMJ articular disc in mammals with lost dentition and corresponding changes in masticatory muscles.^223,224^ In monotremes, such as platypus, a primordial disc is formed but does not mature, and a similar phenotype is observed in mouse mutants with severely reduced cranial musculature due to *Tbx1* deletion in the mesoderm.^225^ As a result, species-specific muscle forces may participate in the evolutionary changes of disc formation in TMJs.

### Cranium

The vertebrate cranium is composed of the cranial vault (or calvaria, including the frontal, parietal, and occipital bones) and the cranial base (including the ethmoid, sphenoid, temporal, and part of the frontal and occipital bones), and together these bones enclose and protect the brain within. The anatomies of the cranium and the brain are well integrated and they accommodate each other in terms of the volumes and the shapes, a result of coordinated growth during development.^226^ While the brain is derived from the neuroectoderm, the cranial bones are derived from mesenchymal cells that originate from either the cranial neural crest (e.g., progenitors for the frontal bone) or the head mesoderm (e.g., progenitors for the parietal bone).^227^ In mouse embryos, these mesenchymal cells begin to condense at E12.5 and form rudiments of frontal and parietal bones above and posterior to the eye, respectively.^228,229^ Next, calvarial rudiments undergo lateral and upward expansion as a result of osteogenic precursors migrating out from the bone primordia.^229,230^ Calvarial bones are then formed through a process known as intramembranous ossification when osteoblasts in the rudiments further differentiate and directly lay down matrices to initiate bone mineralization without going through an intermediate cartilaginous step.^228^ The expanding cranial bones subsequently approach each other. At the site of bone approximation, the opposing osteogenic bone fronts containing osteogenic progenitors and the interposed undifferentiated mesenchymal cells then become the developing suture.^231^

While the brain enlarges in size throughout embryonic and postnatal development, the skull must also expand accordingly. The sutures, as fibrous joints between cranial bones, remain patent (or unfused) during this process and function as an active site for new bone formation that enables skull expansion.^232^ In embryos, this is achieved through maintenance of proliferating osteoprogenitors in the osteogenic bone fronts of the cranial bones, which can generate new osteoblasts and add new bones appositionally.^233^ In postnatal animals, the suture mesenchyme has been shown to retain a group of mesenchymal stem cells expressing *Gli1*, *Prx1*, and *Axin2*, and suture stem cells are responsible for the postnatal growth and turnover of the calvaria, as well as injury repair.^234–236^ Maintaining suture cells in an undifferentiated state is therefore critical for the co-development of the cranium and the brain. Several signaling pathways, including Fgf, BMP, Notch, Ephrin, Wnt, and Hh, are all key regulators in this process and mutations in genes encoding components of the pathways result in pathological fusion of the sutures, or craniosynostosis, disrupting the normal morphology and development of both the cranium and the brain.^237,238^

**Fig. 5:**
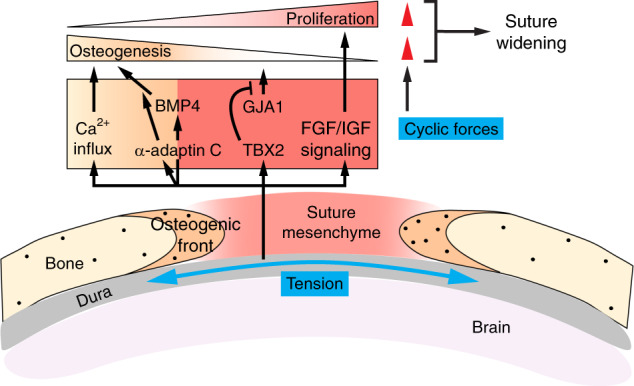
Integration of mechanical and biochemical signals at cranial sutures. In the developing calvaria, mesenchymal cells in the suture midline are proliferative and give rise to osteoprogenitors and osteoblasts in the osteogenic front. The calvaria sits on top of the dura mater and experiences a quasi-static tensile strain (blue arrows) due to the expansion of the growing brain underneath and the intracranial pressure. Such force then signals through FGF and IGF signaling to maintain mesenchymal cell proliferation, as well as TBX2 to inhibit GJA1 and premature differentiation. In the osteogenic front, tensile forces signal through BMP4 and Ca^2+^ influx to promote osteogenesis. α-adaptin C-dependent endocytosis also functions downstream of the tensile stress to promote osteogenic differentiation, possibly by enhancing BMP signals. Cyclic forces generated by masticatory muscle contraction promote both mesenchymal proliferation and osteogenic differentiation (red arrowheads), leading to suture widening

Apart from biochemical signals, it is important to also consider the role of tissue mechanical forces in controlling suture patency and cranial morphology (Fig. 5), given that mechanical signals regulate bone development elsewhere.^239^ Beginning as early as E13 in mice, the calvaria is physically connected to the brain via the dura mater that is part of the meninges. While the dura mater is a source for secreting biochemical ligands to control both ossification and suture patency,^240,241^ it can also in theory relay mechanical forces induced by the increasing brain volume to control the biology of the overlying mesenchymal and bone cells, as originally proposed by Moss.^242^ The idea is that brain enlargement within the confined space of the skull can gradually generate pressure and deform the ECM and cells in the developing cranium, which would experience a tensile strain (mostly quasi-static, or very slow). Indeed, measuring the mouse intracranial pressure showed that the pressure increases with age in postnatal animals between P3 and P70 as brain increases in volume.^243^ A measurable tensile strain is present in both the dura mater and sutures, although that decreases with age presumably due to increased suture stiffness.^244,245^ In human patients with hydrocephalus, excessive shunting of cerebrospinal fluid can cause premature fusion of sutures (synostosis) and this has been postulated to result from reduced intracranial pressure and decreased tensile strain at the suture.^246^ Similarly, synostosis is associated with other pathological conditions, such as microcephaly or intrauterine head constraint,^247^ where sutural strain is also likely diminished. These observations thus indicate that tensile forces may play a role in regulating calvarial development and suture fusion.

Most of our understanding of how tensile forces regulate sutures comes from experiments applying ectopic forces with the use of loaded helical springs to expand sutures in calvaria explants or directly on cranial bones in vivo.^248,249^ These experiments showed that cells in the suture can respond to increased tension and orient themselves in the direction of the force, as well as alter their proliferation and differentiation potential to expand the bones. In this context, sutural mesenchymal cells undergo increased proliferation in response to tension.^250,251^ A corresponding increase in the expression of Insulin-like growth factor I (IGF-1), its receptor, and FGF receptors in midsagittal cells, along with augmented FGF2 protein release from the suture, all indicate that tension may control sutural cell proliferation through IGF and FGF signaling^251–253^ (Fig. 5). Tensile forces also induce TBX2 expression in midsagittal cells, where TBX2 may function to maintain the undifferentiated state of mesenchymal cells and suture patency by inhibiting the expression of the gap junction protein Connexin 43 (GJA1) that normally promotes osteogenic differentiation.^254,255^ Concurrently, tensile strain promotes BMP4 expression in mesenchymal cells and their differentiation towards the osteoblast lineage as evidenced by the increasing number of osteopontin (OPN)-expressing and osteocalcin (OCN)-expressing cells that are recruited to the lengthening osteogenic bone fronts.^250,256,257^ α-adaptin C, a component of the adapter protein 2 (AP-2) complex for clathrin-dependent endocytosis, has also been found to be upregulated in mesenchymal cells and may play a role in modulating signal transduction to promote osteogenic differentiation, as blocking endocytosis suppressed tensile force-induced osteoblast differentiation.^258^ An interesting observation from another study showed that stretching sutures results in an immediate intracellular Ca^2+^ influx.^253^ While the functional significance of Ca^2+^ concentration change remains unclear in this context, Ca^2+^ influx can lead to osteoblast differentiation elsewhere.^259,260^ As conditional deletion of the mechano-sensitive Ca^2+^ ion channel Piezo1 in osteoblasts causes incomplete closure of cranial sutures,^261^ it is intriguing to speculate that tissue forces may signal through Piezo1 and its downstream Ca^2+^-dependent signaling to regulate cell differentiation in sutures. However, the role of endogenous tensile (or compressive) stresses during calvarial and suture development remains unclear and future experiments studying the functional requirement of these forces by means of perturbing force generation or transduction is an important next step.

In addition to the quasi-static strain discussed above, cranial bones are also attached to muscles, which exert forces in a cyclic pattern, such as during feeding. While limited data suggest that muscle forces are dispensable for the formation of sutures during embryonic development (and thus different from synovial joints, like TMJ, on that aspect),^262^ muscle loading in postnatal animals can modulate suture morphology and its interdigitation patterns. For instance, surgical excision of temporal muscles can cause reduced complexity of the sagittal suture interdigitations in rats.^263^ Animals applying less masticatory forces, either from eating a soft diet or due to absence of tooth eruption, also develop structurally simpler sutures and sometimes with synostosis.^264,265^ On the contrary, when bite forces increase, such as in the *Gdf-8* null mice that have lost the myogenesis inhibitor myostatin and form significantly enlarged jaw muscles, there is increased suture complexity.^266^ In the same mutants, age-dependent changes in cranial vault morphology have also been observed, suggesting that muscle forces can remodel calvarial bones.^267,268^ To specifically study the effects of cyclic forces on cranial development, a series of experiments were conducted by applying ectopic cyclic tensile or compressive forces to animals for a period each day.^269–274^ When comparing to sham controls and animals receiving static loading, cyclic forces, regardless of tension or compression, induce suture widening, increased number of suture cells, and heightened osteogenesis.^269,273^ Cyclic forces also trigger expression of matrix metalloproteases MMP-1 and MMP-2 at the suture, which are important for bone mineralization, as well as craniofacial and suture development.^271,275^ Interestingly, suture cells isolated from neonatal rats are mechanosensitive to cyclic tension in culture and display increased osteogenesis with upregulated RUNX2 and OPN expression.^276^ This mechanically-induced osteogenic differentiation program depends on ROCK activity, which promotes nuclear TAZ localization and its subsequent activation of *Runx2* expression. It is conceivable that the same mechanotransduction pathway may be responsible in mediating mechanical signals in vivo to regulate suture osteogenesis. In addition, because actomyosin tension and TAZ localization can be modulated by substrate stiffness,^104^ and stiffer substrate promotes suture cell differentiation,^277^ it will be important in the future to understand how tissue forces remodel suture ECM compositions and stiffness and how changes in suture material properties modulate signaling changes, including those mediated by the Hippo pathway and Piezo ion channels, to control suture cell differentiation.

## Conclusion

While there has been progress in understanding the role of mechanical inputs in regulating the development of various craniofacial structures, many outstanding questions remain. For example, how do cells sense and transduce mechanical information to regulate gene expression? In addition to YAP and Piezo proteins, forces transmitted through cytoskeletons and nuclear membrane complexes can directly deform the nucleus and impact chromatin organizations.^278^ Given that mutations in several nuclear envelop proteins, such as lamins, can cause craniofacial defects,^279,280^ it will be important to investigate the role of nuclear mechanotransduction during craniofacial development. Furthermore, what are the signaling cues that induce mechanical anisotropy and inhomogeneity during organ development? What is the feedback mechanism that modulates the amount and direction of forces at both cellular and tissue levels to achieve adequate shape changes? How are mechanical signals regulated differently to generate diverse morphologies across different species and during evolution? By integrating genetic and biochemical approaches with novel biomechanical techniques, such as oil microdroplets and magnetic beads to quantify absolute force magnitudes and apply forces locally,^81,281,282^ we are closer than ever to address these questions. A deeper understanding of the mechanical control of craniofacial morphogenesis and development will ultimately contribute to novel strategies for manipulating organ-specific progenitor cells, bioengineering tissues with the correct shape and architecture, and advancing stem cell-based regenerative therapies that will transform patient treatment.

## References

[1] Opitz JM (2004). Goethe’s bone and the beginnings of morphology. Am. J. Med. Genet. A..

[2] Thompson, D. W. & Thompson, D. W. *On Growth and Form*. (Cambridge University Press, 1917).

[3] Kindberg A, Hu JK, Bush JO (2020). Forced to communicate: Integration of mechanical and biochemical signaling in morphogenesis. Curr. Opin. Cell Biol..

[4] Stooke-Vaughan GA, Campàs O (2018). Physical control of tissue morphogenesis across scales. Curr. Opin. Genet. Dev..

[5] Huycke TR, Eames BF, Kimmel CB (2012). Hedgehog-dependent proliferation drives modular growth during morphogenesis of a dermal bone. Development.

[6] Wu P, Jiang T-X, Suksaweang S, Widelitz RB, Chuong C-M (2004). Molecular shaping of the beak. Science.

[7] Shraiman BI (2005). Mechanical feedback as a possible regulator of tissue growth. Proc. Natl Acad. Sci. USA.

[8] Mao Y (2013). Differential proliferation rates generate patterns of mechanical tension that orient tissue growth. EMBO J..

[9] Aegerter-Wilmsen T, Aegerter CM, Hafen E, Basler K (2007). Model for the regulation of size in the wing imaginal disc of Drosophila. Mech. Dev..

[10] Aegerter-Wilmsen T (2010). Exploring the effects of mechanical feedback on epithelial topology. Development.

[11] Streichan SJ, Hoerner CR, Schneidt T, Holzer D, Hufnagel L (2014). Spatial constraints control cell proliferation in tissues. Proc. Natl Acad. Sci. USA.

[12] Pan Y, Heemskerk I, Ibar C, Shraiman BI, Irvine KD (2016). Differential growth triggers mechanical feedback that elevates Hippo signaling. Proc. Natl Acad. Sci. USA.

[13] Bornhorst D (2019). Biomechanical signaling within the developing zebrafish heart attunes endocardial growth to myocardial chamber dimensions. Nat. Commun..

[14] Gros J, Scaal M, Marcelle C (2004). A two-step mechanism for myotome formation in chick. Dev. Cell.

[15] Wyngaarden LA (2010). Oriented cell motility and division underlie early limb bud morphogenesis. Dev. Camb. Engl..

[16] Baena-López LA, Baonza A, García-Bellido A (2005). The orientation of cell divisions determines the shape of drosophila organs. Curr. Biol..

[17] Wyatt TPJ (2015). Emergence of homeostatic epithelial packing and stress dissipation through divisions oriented along the long cell axis. Proc. Natl Acad. Sci. USA.

[18] Zhou Z, Alégot H, Irvine KD (2019). Oriented cell divisions are not required for drosophila wing shape. Curr. Biol..

[19] Campinho P (2013). Tension-oriented cell divisions limit anisotropic tissue tension in epithelial spreading during zebrafish epiboly. Nat. Cell Biol..

[20] Boehm B (2010). The role of spatially controlled cell proliferation in limb bud morphogenesis. PLoS Biol..

[21] Tao H (2019). Oscillatory cortical forces promote three dimensional cell intercalations that shape the murine mandibular arch. Nat. Commun..

[22] Footer MJ, Kerssemakers JWJ, Theriot JA, Dogterom M (2007). Direct measurement of force generation by actin filament polymerization using an optical trap. Proc. Natl Acad. Sci. USA.

[23] Dogterom M, Yurke B (1997). Measurement of the force-velocity relation for growing microtubules. Science.

[24] Vicente-Manzanares M, Ma X, Adelstein RS, Horwitz AR (2009). Non-muscle myosin II takes centre stage in cell adhesion and migration. Nat. Rev. Mol. Cell Biol..

[25] Munjal A, Lecuit T (2014). Actomyosin networks and tissue morphogenesis. Development.

[26] Agarwal P, Zaidel-Bar R (2019). Principles of actomyosin regulation in vivo. Trends Cell Biol..

[27] Martin AC, Kaschube M, Wieschaus EF (2009). Pulsed contractions of an actin-myosin network drive apical constriction. Nature.

[28] Mason FM, Tworoger M, Martin AC (2013). Apical domain polarization localizes actin-myosin activity to drive ratchet-like apical constriction. Nat. Cell Biol..

[29] Borges RM, Lamers ML, Forti FL, Santos MFD, Yan CYI (2011). Rho signaling pathway and apical constriction in the early lens placode. Genes. N. Y. N..

[30] Sai X, Yonemura S, Ladher RK (2014). Junctionally restricted RhoA activity is necessary for apical constriction during phase 2 inner ear placode invagination. Dev. Biol..

[31] Zhang S, Lee J-M, Ashok AA, Jung H-S (2020). Action of actomyosin contraction with shh modulation drive epithelial folding in the circumvallate papilla. Front. Physiol.

[32] Kasza KE, Farrell DL, Zallen JA (2014). Spatiotemporal control of epithelial remodeling by regulated myosin phosphorylation. Proc. Natl Acad. Sci. USA.

[33] Simões S (2010). Rho-kinase directs Bazooka/Par-3 planar polarity during Drosophila axis elongation. Dev. Cell.

[34] Rauzi M, Lenne P-F, Lecuit T (2010). Planar polarized actomyosin contractile flows control epithelial junction remodelling. Nature.

[35] Bertet C, Sulak L, Lecuit T (2004). Myosin-dependent junction remodelling controls planar cell intercalation and axis elongation. Nature.

[36] Heisenberg CP (2000). Silberblick/Wnt11 mediates convergent extension movements during zebrafish gastrulation. Nature.

[37] Nishimura T, Honda H, Takeichi M (2012). Planar cell polarity links axes of spatial dynamics in neural-tube closure. Cell.

[38] Keller R (2002). Shaping the vertebrate body plan by polarized embryonic cell movements. Science.

[39] Harris TJC, Tepass U (2010). Adherens junctions: from molecules to morphogenesis. Nat. Rev. Mol. Cell Biol..

[40] le Duc Q (2010). Vinculin potentiates E-cadherin mechanosensing and is recruited to actin-anchored sites within adherens junctions in a myosin II-dependent manner. J. Cell Biol..

[41] Yonemura S, Wada Y, Watanabe T, Nagafuchi A, Shibata M (2010). α-Catenin as a tension transducer that induces adherens junction development. Nat. Cell Biol..

[42] Leerberg JM (2014). Tension-sensitive actin assembly supports contractility at the epithelial zonula adherens. Curr. Biol. CB.

[43] Choi H-J (2012). αE-catenin is an autoinhibited molecule that coactivates vinculin. Proc. Natl Acad. Sci. USA.

[44] Yao M (2014). Force-dependent conformational switch of α-catenin controls vinculin binding. Nat. Commun..

[45] Shewan AM (2005). Myosin 2 is a key rho kinase target necessary for the local concentration of E-cadherin at cell–cell contacts. Mol. Biol. Cell.

[46] Verma S (2012). A WAVE2-Arp2/3 actin nucleator apparatus supports junctional tension at the epithelial zonula adherens. Mol. Biol. Cell.

[47] Kale GR (2018). Distinct contributions of tensile and shear stress on E-cadherin levels during morphogenesis. Nat. Commun..

[48] Gittes F, Mickey B, Nettleton J, Howard J (1993). Flexural rigidity of microtubules and actin filaments measured from thermal fluctuations in shape. J. Cell Biol..

[49] Wang N (2001). Mechanical behavior in living cells consistent with the tensegrity model. Proc. Natl Acad. Sci. USA.

[50] Singh A (2018). Polarized microtubule dynamics directs cell mechanics and coordinates forces during epithelial morphogenesis. Nat. Cell Biol..

[51] Takeda M, Sami MM, Wang Y-C (2018). A homeostatic apical microtubule network shortens cells for epithelial folding via a basal polarity shift. Nat. Cell Biol..

[52] Stehbens SJ (2006). Dynamic microtubules regulate the local concentration of E-cadherin at cell-cell contacts. J. Cell Sci..

[53] Sumigray KD, Foote HP, Lechler T (2012). Noncentrosomal microtubules and type II myosins potentiate epidermal cell adhesion and barrier formation. J. Cell Biol..

[54] Isrie M (2015). Mutations in either TUBB or MAPRE2 cause circumferential skin creases kunze type. Am. J. Hum. Genet..

[55] Larson TA, Gordon TN, Lau HE, Parichy DM (2010). Defective adult oligodendrocyte and Schwann cell development, pigment pattern, and craniofacial morphology in puma mutant zebrafish having an alpha tubulin mutation. Dev. Biol..

[56] Joo EE, Yamada KM (2014). MYPT1 regulates contractility and microtubule acetylation to modulate integrin adhesions and matrix assembly. Nat. Commun..

[57] Short KM, Hopwood B, Yi Z, Cox TC (2002). MID1 and MID2 homo- and heterodimerise to tether the rapamycin-sensitive PP2A regulatory subunit, Alpha 4, to microtubules: implications for the clinical variability of X-linked Opitz GBBB syndrome and other developmental disorders. BMC Cell Biol..

[58] Shwartz Y, Blitz E, Zelzer E (2013). One load to rule them all: mechanical control of the musculoskeletal system in development and aging. Differentiation.

[59] Hall BK, Herring SW (1990). Paralysis and growth of the musculoskeletal system in the embryonic chick. J. Morphol..

[60] Hogg DA, Hosseini A (1992). The effects of paralysis on skeletal development in the chick embryo. Comp. Biochem. Physiol. A Physiol..

[61] Sharir A, Stern T, Rot C, Shahar R, Zelzer E (2011). Muscle force regulates bone shaping for optimal load-bearing capacity during embryogenesis. Development.

[62] Pai AC (1965). Developmental genetics of a lethal mutation, muscular dysgenesis (mdg), in the mouse: I. Genetic analysis and gross morphology. Dev. Biol..

[63] Rot-Nikcevic I (2006). Myf5−/−:MyoD−/−amyogenic fetuses reveal the importance of early contraction and static loading by striated muscle in mouse skeletogenesis. Dev. Genes Evol..

[64] Blitz E (2009). Bone ridge patterning during musculoskeletal assembly is mediated through SCX regulation of Bmp4 at the tendon-skeleton junction. Dev. Cell.

[65] Maeda T (2011). Conversion of mechanical force into TGF-β-mediated biochemical signals. Curr. Biol..

[66] Huang AH (2015). Musculoskeletal integration at the wrist underlies the modular development of limb tendons. Dev. Camb. Engl..

[67] Kahn J (2009). Muscle contraction is necessary to maintain joint progenitor cell fate. Dev. Cell.

[68] Solem RC, Eames BF, Tokita M, Schneider RA (2011). Mesenchymal and mechanical mechanisms of secondary cartilage induction. Dev. Biol..

[69] Shwartz Y, Farkas Z, Stern T, Aszódi A, Zelzer E (2012). Muscle contraction controls skeletal morphogenesis through regulation of chondrocyte convergent extension. Dev. Biol..

[70] Subramanian A, Kanzaki LF, Galloway JL, Schilling TF (2018). Mechanical force regulates tendon extracellular matrix organization and tenocyte morphogenesis through TGFbeta signaling. eLife.

[71] Jones DC, Zelditch ML, Peake PL, German RZ (2007). The effects of muscular dystrophy on the craniofacial shape of Mus musculus. J. Anat..

[72] Lightfoot PS, German RZ (1998). The effects of muscular dystrophy on craniofacial growth in mice: a study of heterochrony and ontogenetic allometry. J. Morphol..

[73] Matsuyuki T, Kitahara T, Nakashima A (2006). Developmental changes in craniofacial morphology in subjects with Duchenne muscular dystrophy. Eur. J. Orthod..

[74] Wong M, Siegrist M, Goodwin K (2003). Cyclic tensile strain and cyclic hydrostatic pressure differentially regulate expression of hypertrophic markers in primary chondrocytes. Bone.

[75] Mikic B, Isenstein AL, Chhabra A (2004). Mechanical modulation of cartilage structure and function during embryogenesis in the chick. Ann. Biomed. Eng..

[76] Luu O, David R, Ninomiya H, Winklbauer R (2011). Large-scale mechanical properties of Xenopus embryonic epithelium. Proc. Natl Acad. Sci.USA.

[77] Zhou J, Kim HY, Davidson LA (2009). Actomyosin stiffens the vertebrate embryo during crucial stages of elongation and neural tube closure. Dev. Camb. Engl..

[78] Shawky JH, Balakrishnan UL, Stuckenholz C, Davidson LA (2018). Multiscale analysis of architecture, cell size and the cell cortex reveals cortical F-actin density and composition are major contributors to mechanical properties during convergent extension. Development.

[79] Iyer KV, Piscitello-Gómez R, Paijmans J, Jülicher F, Eaton S (2019). Epithelial viscoelasticity is regulated by mechanosensitive E-cadherin turnover. Curr. Biol. CB.

[80] Serwane F (2017). In vivo quantification of spatially varying mechanical properties in developing tissues. Nat. Methods.

[81] Mongera A (2018). A fluid-to-solid jamming transition underlies vertebrate body axis elongation. Nature.

[82] Bénazéraf B (2010). A random cell motility gradient downstream of FGF controls elongation of an amniote embryo. Nature.

[83] Dzamba, B. J. & DeSimone, D. W. Extracellular matrix (ECM) and the sculpting of embryonic tissues. In *Current Topics in Developmental Biology* (eds. Litscher, E. S. & Wassarman, P. M.) Vol. 130, 245–274 (Academic Press, 2018). 10.1016/bs.ctdb.2018.03.006 29853179

[84] del Rio A (2009). Stretching single talin rod molecules activates vinculin binding. Science.

[85] Yao M (2014). Mechanical activation of vinculin binding to talin locks talin in an unfolded conformation. Sci. Rep..

[86] Muncie, J. M. & Weaver, V. M. The physical and biochemical properties of the extracellular matrix regulate cell fate. In *Current Topics in Developmental Biology* (eds. Litscher, E. S. & Wassarman, P. M.) Vol. 130, 1–37 (Academic Press, 2018). 10.1016/bs.ctdb.2018.02.002 PMC6586474 29853174

[87] Nakanishi Y, Sugiura F, Kishi J, Hayakawa T (1986). Collagenase inhibitor stimulates cleft formation during early morphogenesis of mouse salivary gland. Dev. Biol..

[88] Sakai T, Larsen M, Yamada KM (2003). Fibronectin requirement in branching morphogenesis. Nature.

[89] Daley WP, Gulfo KM, Sequeira SJ, Larsen M (2009). Identification of a mechanochemical checkpoint and negative feedback loop regulating branching morphogenesis. Dev. Biol..

[90] Daley WP, Kohn JM, Larsen M (2011). A focal adhesion protein-based mechanochemical checkpoint regulates cleft progression during branching morphogenesis. Dev. Dyn..

[91] Barriga EH, Franze K, Charras G, Mayor R (2018). Tissue stiffening coordinates morphogenesis by triggering collective cell migration in vivo. Nature.

[92] Thesleff I, Vainio S, Jalkanen M (1989). Cell-matrix interactions in tooth development. Int. J. Dev. Biol..

[93] Mammoto Tadanori (2015). Mesenchymal condensation‐dependent accumulation of collagen VI stabilizes organ‐specific cell fates during embryonic tooth formation. Dev. Dyn..

[94] Huang J (2011). The mechanism of lens placode formation: a case of matrix-mediated morphogenesis. Dev. Biol..

[95] Bogdanović O (2012). Numb/Numbl-Opo antagonism controls retinal epithelium morphogenesis by regulating integrin endocytosis. Dev. Cell.

[96] Visconti RP, Hilfer SR (2002). Perturbation of extracellular matrix prevents association of the otic primordium with the posterior rhombencephalon and inhibits subsequent invagination. Dev. Dyn..

[97] Geng F-S (2013). Semicircular canal morphogenesis in the zebrafish inner ear requires the function of gpr126 (lauscher), an adhesion class G protein-coupled receptor gene. Development.

[98] Croucher SJ, Tickle C (1989). Characterization of epithelial domains in the nasal passages of chick embryos: spatial and temporal mapping of a range of extracellular matrix and cell surface molecules during development of the nasal placode. Dev. Camb. Engl..

[99] Karaman R, Halder G (2018). Cell junctions in Hippo signaling. Cold Spring Harb. Perspect. Biol..

[100] Schlegelmilch K (2011). Yap1 acts downstream of α-catenin to control epidermal proliferation. Cell.

[101] Zhao B (2011). Angiomotin is a novel Hippo pathway component that inhibits YAP oncoprotein. Genes Dev..

[102] Feng X (2019). A platform of synthetic lethal gene interaction networks reveals that the GNAQ uveal melanoma oncogene controls the Hippo pathway through FAK. Cancer Cell.

[103] Li P (2016). αE-catenin inhibits a Src-YAP1 oncogenic module that couples tyrosine kinases and the effector of Hippo signaling pathway. Genes Dev..

[104] Dupont S (2011). Role of YAP/TAZ in mechanotransduction. Nature.

[105] Elosegui-Artola A (2016). Mechanical regulation of a molecular clutch defines force transmission and transduction in response to matrix rigidity. Nat. Cell Biol..

[106] Kim N-G, Gumbiner BM (2015). Adhesion to fibronectin regulates Hippo signaling via the FAK-Src-PI3K pathway. J. Cell Biol..

[107] Elosegui-Artola A (2017). Force triggers YAP nuclear entry by regulating transport across nuclear pores. Cell.

[108] Rauskolb C, Sun S, Sun G, Pan Y, Irvine KD (2014). Cytoskeletal tension inhibits Hippo signaling through an Ajuba-Warts complex. Cell.

[109] Ibar C (2018). Tension-dependent regulation of mammalian Hippo signaling through LIMD1. J. Cell Sci..

[110] Wang J (2016). Yap and Taz play a crucial role in neural crest-derived craniofacial development. Development.

[111] Liu M, Zhao S, Lin Q, Wang X-P (2015). YAP regulates the expression of Hoxa1 and Hoxc13 in mouse and human oral and skin epithelial tissues. Mol. Cell. Biol..

[112] Goodwin, A. F., Chen, C. P., Vo, N. T., Bush, J. O. & Klein, O. D. YAP/TAZ regulate elevation and bone formation of the mouse secondary palate. *J. Dent. Res.* 0022034520935372 10.1177/0022034520935372. (2020) 10.1177/0022034520935372 PMC7580170 32623954

[113] McMillin MJ (2014). Mutations in PIEZO2 cause Gordon syndrome, Marden-Walker syndrome, and distal arthrogryposis type 5. Am. J. Hum. Genet..

[114] Wu J, Lewis AH, Grandl J (2017). Touch, tension, and transduction–the function and regulation of piezo ion channels. Trends Biochem. Sci..

[115] Eisenhoffer GT (2012). Crowding induces live cell extrusion to maintain homeostatic cell numbers in epithelia. Nature.

[116] Pathak MM (2014). Stretch-activated ion channel Piezo1 directs lineage choice in human neural stem cells. Proc. Natl Acad. Sci. USA.

[117] He L, Si G, Huang J, Samuel ADT, Perrimon N (2018). Mechanical regulation of stem-cell differentiation by the stretch-activated Piezo channel. Nature.

[118] Okubo T (2011). Ripply3, a Tbx1 repressor, is required for development of the pharyngeal apparatus and its derivatives in mice. Development.

[119] Frisdal A, Trainor PA (2014). Development and evolution of the pharyngeal apparatus. Wiley Interdiscip. Rev. Dev. Biol..

[120] Wilkie AOM, Morriss-Kay GM (2001). Genetics of craniofacial development and malformation. Nat. Rev. Genet..

[121] Trumpp A, Depew MJ, Rubenstein JL, Bishop JM, Martin GR (1999). Cre-mediated gene inactivation demonstrates that FGF8 is required for cell survival and patterning of the first branchial arch. Genes Dev..

[122] Berge Dten (2001). Prx1 and Prx2 are upstream regulators of sonic hedgehog and control cell proliferation during mandibular arch morphogenesis. Development.

[123] Ota MS (2004). Twist is required for patterning the cranial nerves and maintaining the viability of mesodermal cells. Dev. Dyn..

[124] Yamaguchi TP, Bradley A, McMahon AP, Jones S (1999). A Wnt5a pathway underlies outgrowth of multiple structures in the vertebrate embryo. Development.

[125] Person AD (2010). WNT5A mutations in patients with autosomal dominant Robinow syndrome. Dev. Dyn..

[126] Gros J (2010). WNT5A/JNK and FGF/MAPK pathways regulate the cellular events shaping the vertebrate limb bud. Curr. Biol..

[127] Hosseini-Farahabadi, S., Gignac, S. J., Danescu, A., Fu, K. & Richman, J. M. Abnormal WNT5A signaling causes mandibular hypoplasia in Robinow syndrome. *J. Dent. Res*. 10.1177/0022034517716916. (2017). 10.1177/0022034517716916 28662348

[128] Zhu M, Zhang K, Tao H, Hopyan S, Sun Y (2020). Magnetic micromanipulation for in vivo measurement of stiffness heterogeneity and anisotropy in the mouse mandibular arch. Research.

[129] Smith MM (2003). Vertebrate dentitions at the origin of jaws: when and how pattern evolved. Evol. Dev..

[130] Thesleff I, Nieminen P (1996). Tooth morphogenesis and cell differentiation. Curr. Opin. Cell Biol..

[131] Yu T, Klein OD (2020). Molecular and cellular mechanisms of tooth development, homeostasis and repair. Development.

[132] Kim R, Green JBA, Klein OD (2017). From snapshots to movies: understanding early tooth development in four dimensions. Dev. Dyn..

[133] Panousopoulou E, Green JBA (2016). Invagination of ectodermal placodes is driven by cell intercalation-mediated contraction of the suprabasal tissue canopy. PLoS Biol..

[134] Pearl EJ, Li J, Green JBA (2017). Cellular systems for epithelial invagination. Philos. Trans. R. Soc. Lond. B.

[135] Li J, Economou AD, Vacca B, Green JBA (2020). Epithelial invagination by a vertical telescoping cell movement in mammalian salivary glands and teeth. Nat. Commun..

[136] Li J, Chatzeli L, Panousopoulou E, Tucker AS, Green JBA (2016). Epithelial stratification and placode invagination are separable functions in early morphogenesis of the molar tooth. Dev. Camb. Engl..

[137] Mammoto T (2011). Mechanochemical control of mesenchymal condensation and embryonic tooth organ formation. Dev. Cell.

[138] Butler PM (1962). Distribution of mitoses in the inner enamel epithelium of molar tooth germs of the mouse. J. Dent. Res..

[139] Jernvall J, Aberg T, Kettunen P, Keränen S, Thesleff I (1998). The life history of an embryonic signaling center: BMP-4 induces p21 and is associated with apoptosis in the mouse tooth enamel knot. Dev. Camb. Engl..

[140] Vaahtokari A, Åberg T, Jernvall J, Keränen S, Thesleff I (1996). The enamel knot as a signaling center in the developing mouse tooth. Mech. Dev..

[141] Salazar-Ciudad I, Jernvall J (2010). A computational model of teeth and the developmental origins of morphological variation. Nature.

[142] Morita R (2016). Coordination of cellular dynamics contributes to tooth epithelium deformations. PLoS ONE.

[143] Takigawa-Imamura H, Morita R, Iwaki T, Tsuji T, Yoshikawa K (2015). Tooth germ invagination from cell-cell interaction: Working hypothesis on mechanical instability. J. Theor. Biol..

[144] Marin-Riera M, Moustakas-Verho J, Savriama Y, Jernvall J, Salazar-Ciudad I (2018). Differential tissue growth and cell adhesion alone drive early tooth morphogenesis: an ex vivo and in silico study. PLoS Comput. Biol..

[145] Yamada S, Lav R, Li J, Tucker AS, Green JBA (2019). Molar bud-to-cap transition is proliferation independent. J. Dent. Res..

[146] Li C-Y (2016). E-catenin inhibits YAP/TAZ activity to regulate signalling centre formation during tooth development. Nat. Commun..

[147] Jernvall, J., Keränen, S. V. E. & Thesleff, I. Evolutionary modification of development in mammalian teeth: quantifying gene expression patterns and topography. *Proc. Natl Acad. Sci. USA*10.1073/pnas.97.26.14444. (2000). 10.1073/pnas.97.26.14444 PMC18938 11121045

[148] Coin R, Lesot H, Vonesch JL, Haikel Y, Ruch JV (1999). Aspects of cell proliferation kinetics of the inner dental epithelium during mouse molar and incisor morphogenesis: a reappraisal of the role of the enamel knot area. Int. J. Dev. Biol..

[149] Du W, Hu JK-H, Du W, Klein OD (2017). Lineage tracing of epithelial cells in developing teeth reveals two strategies for building signaling centers. J. Biol. Chem..

[150] Jernvall J, Thesleff I (2012). Tooth shape formation and tooth renewal: evolving with the same signals. Development.

[151] Renvoisé E (2017). Mechanical constraint from growing jaw facilitates mammalian dental diversity. Proc. Natl Acad. Sci. USA.

[152] Wu X (2020). Biomechanical stress regulates mammalian tooth replacement via the integrin β1-RUNX2-Wnt pathway. EMBO J..

[153] Smith EE (2017). Developing a biomimetic tooth bud model. J. Tissue Eng. Regen. Med..

[154] Brown TE, Anseth KS (2017). Spatiotemporal hydrogel biomaterials for regenerative medicine. Chem. Soc. Rev..

[155] Krueger D (2019). Principles and applications of optogenetics in developmental biology. Development.

[156] Marrelli M (2018). Dental pulp stem cell mechanoresponsiveness: effects of mechanical stimuli on dental pulp stem cell behavior. Front. Physiol..

[157] Zhang R, Wan J, Wang H (2019). Mechanical strain triggers differentiation of dental mesenchymal stem cells by activating osteogenesis-specific biomarkers expression. Am. J. Transl. Res..

[158] Ito Y (2003). Conditional inactivation of Tgfbr2 in cranial neural crest causes cleft palate and calvaria defects. Development.

[159] Bush JO, Jiang R (2012). Palatogenesis: morphogenetic and molecular mechanisms of secondary palate development. Development.

[160] Dixon MJ, Marazita ML, Beaty TH, Murray JC (2011). Cleft lip and palate: understanding genetic and environmental influences. Nat. Rev. Genet..

[161] Kaartinen V (1995). Abnormal lung development and cleft palate in mice lacking TGF-beta 3 indicates defects of epithelial-mesenchymal interaction. Nat. Genet..

[162] Ingraham CR (2006). Abnormal skin, limb and craniofacial morphogenesis in mice deficient for interferon regulatory factor 6 (Irf6). Nat. Genet..

[163] Richardson RJ (2006). Irf6 is a key determinant of the keratinocyte proliferation-differentiation switch. Nat. Genet..

[164] Ke C-Y, Xiao W-L, Chen C-M, Lo L-J, Wong F-H (2015). IRF6 is the mediator of TGFβ3 during regulation of the epithelial mesenchymal transition and palatal fusion. Sci. Rep..

[165] Vieira AR (2008). Unraveling human cleft lip and palate research. J. Dent. Res..

[166] Kondo S (2002). Mutations in IRF6 cause Van der Woude and popliteal pterygium syndromes. Nat. Genet..

[167] Jin J-Z (2010). Mesenchymal cell remodeling during mouse secondary palate reorientation. Dev. Dyn..

[168] Yu K, Ornitz DM (2011). Histomorphological study of palatal shelf elevation during murine secondary palate formation. Dev. Dyn..

[169] Brock LJ, Economou AD, Cobourne MT, Green JBA (2016). Mapping cellular processes in the mesenchyme during palatal development in the absence of Tbx1 reveals complex proliferation changes and perturbed cell packing and polarity. J. Anat..

[170] Lan Y (2004). Odd-skipped related 2 (Osr2) encodes a key intrinsic regulator of secondary palate growth and morphogenesis. Development.

[171] Chiquet M, Blumer S, Angelini M, Mitsiadis TA, Katsaros C (2016). Mesenchymal remodeling during palatal shelf elevation revealed by extracellular matrix and F-actin expression patterns. Front. Physiol..

[172] Wang X (2020). Extracellular matrix remodeling during palate development. Organogenesis.

[173] Wang C (2013). Type 1 fibroblast growth factor receptor in cranial neural crest cell-derived mesenchyme is required for palatogenesis. J. Biol. Chem..

[174] Pratt RM, Goggins JF, Wilk AL, King CT (1973). Acid mucopolysaccharide synthesis in the secondary palate of the developing rat at the time of rotation and fusion. Dev. Biol..

[175] Ferguson MW (1988). Palate development. Dev. Camb. Engl..

[176] Galloway JL, Jones SJ, Mossey PA, Ellis IR (2013). The control and importance of hyaluronan synthase expression in palatogenesis. Front. Physiol..

[177] Lan Y, Qin C, Jiang R (2019). Requirement of hyaluronan synthase-2 in craniofacial and palate development. J. Dent. Res..

[178] Yonemitsu MA, Lin T, Yu K (2020). Hyaluronic acid is required for palatal shelf movement and its interaction with the tongue during palatal shelf elevation. Dev. Biol..

[179] Yu K, Yonemitsu MA (2019). In vitro analysis of palatal shelf elevation during secondary palate formation. Anat. Rec. Hoboken NJ.

[180] Zhang J (2015). Loss of lysyl oxidase-like 3 causes cleft palate and spinal deformity in mice. Hum. Mol. Genet..

[181] Vanyai, H. K. et al. Control of skeletal morphogenesis by the Hippo-YAP/TAZ pathway. *Development*10.1242/dev.187187. (2020). 10.1242/dev.187187 PMC7673359 32994166

[182] Fitchett JE, Hay ED (1989). Medial edge epithelium transforms to mesenchyme after embryonic palatal shelves fuse. Dev. Biol..

[183] Griffith CM, Hay ED (1992). Epithelial-mesenchymal transformation during palatal fusion: carboxyfluorescein traces cells at light and electron microscopic levels. Dev. Camb. Engl..

[184] Shuler CF, Guo Y, Majumder A, Luo RY (1991). Molecular and morphologic changes during the epithelial-mesenchymal transformation of palatal shelf medial edge epithelium in vitro. Int. J. Dev. Biol..

[185] Martínez-Alvarez C (2000). Medial edge epithelial cell fate during palatal fusion. Dev. Biol..

[186] DeAngelis V, Nalbandian J (1968). Ultrastructure of mouse and rat palatal processes prior to and during secondary palate formation. Arch. Oral. Biol..

[187] Cuervo R, Valencia C, Chandraratna RAS, Covarrubias L (2002). Programmed cell death is required for palate shelf fusion and is regulated by retinoic acid. Dev. Biol..

[188] Cuervo R, Covarrubias L (2004). Death is the major fate of medial edge epithelial cells and the cause of basal lamina degradation during palatogenesis. Development.

[189] Carette MJ, Ferguson MW (1992). The fate of medial edge epithelial cells during palatal fusion in vitro: an analysis by DiI labelling and confocal microscopy. Dev. Camb. Engl..

[190] Jin J-Z, Ding J (2006). Analysis of cell migration, transdifferentiation and apoptosis during mouse secondary palate fusion. Development.

[191] Lohnes D (1994). Function of the retinoic acid receptors (RARs) during development (I). Craniofacial and skeletal abnormalities in RAR double mutants. Dev. Camb. Engl..

[192] Richardson RJ, Dixon J, Jiang R, Dixon MJ (2009). Integration of IRF6 and Jagged2 signalling is essential for controlling palatal adhesion and fusion competence. Hum. Mol. Genet..

[193] Iwata J (2013). Smad4-Irf6 genetic interaction and TGFβ-mediated IRF6 signaling cascade are crucial for palatal fusion in mice. Dev. Camb. Engl..

[194] Lane J (2015). Tak1, Smad4 and Trim33 redundantly mediate TGF-β3 signaling during palate development. Dev. Biol..

[195] Ke FFS (2018). Embryogenesis and adult life in the absence of intrinsic apoptosis effectors BAX, BAK, and BOK. Cell.

[196] Kim S (2015). Convergence and extrusion are required for normal fusion of the mammalian secondary palate. PLoS Biol..

[197] Li, J. et al. Linking suckling biomechanics to the development of the palate. *Sci. Rep.* **6**, (2016). 10.1038/srep20419 PMC4740798 26842915

[198] Yuan Y, Chai Y (2019). Regulatory mechanisms of jaw bone and tooth development. Curr. Top. Dev. Biol..

[199] Ivkovic S (2003). Connective tissue growth factor coordinates chondrogenesis and angiogenesis during skeletal development. Development.

[200] Mori-Akiyama Y, Akiyama H, Rowitch DH, de Crombrugghe B (2003). Sox9 is required for determination of the chondrogenic cell lineage in the cranial neural crest. Proc. Natl Acad. Sci. USA.

[201] Wolff, J. *The Law of Bone Remodelling* (Translation of the original German edition). (Springer-Verlag, 1892).

[202] Habib H, Hatta T, Rahman OIF, Yoshimura Y, Otani H (2007). Fetal jaw movement affects development of articular disk in the temporomandibular joint. Congenit. Anom..

[203] Habib H (2005). Fetal jaw movement affects condylar cartilage development. J. Dent. Res..

[204] Jahan E (2014). Fetal jaw movement affects Ihh signaling in mandibular condylar cartilage development: the possible role of Ihh as mechanotransduction mediator. Arch. Oral. Biol..

[205] Wu Q, Zhang Y, Chen Q (2001). Indian hedgehog is an essential component of mechanotransduction complex to stimulate chondrocyte proliferation. J. Biol. Chem..

[206] Brunt LH, Norton JL, Bright JA, Rayfield EJ, Hammond CL (2015). Finite element modelling predicts changes in joint shape and cell behaviour due to loss of muscle strain in jaw development. J. Biomech..

[207] Brunt LH (2016). Differential effects of altered patterns of movement and strain on joint cell behaviour and skeletal morphogenesis. Osteoarthr. Cartil..

[208] Brunt LH, Begg K, Kague E, Cross S, Hammond CL (2017). Wnt signalling controls the response to mechanical loading during zebrafish joint development. Development.

[209] Sella-Tunis T, Pokhojaev A, Sarig R, O’Higgins P, May H (2018). Human mandibular shape is associated with masticatory muscle force. Sci. Rep..

[210] Kiliaridis S, Mejersjö C, Thilander B (1989). Muscle function and craniofacial morphology: a clinical study in patients with myotonic dystrophy. Eur. J. Orthod..

[211] Hassan MG (2020). Effects of multi-generational soft diet consumption on mouse craniofacial morphology. Front. Physiol.

[212] Simon MR (1977). The role of compressive forces in the normal maturation of the condylar cartilage in the rat. Cells Tissues Organs.

[213] Hinton RJ, Carlson DS (1986). Response of the MAndibular Joint to Loss of Incisal Function in the Rat. Cells Tissues Organs.

[214] Kantomaa T, Tuominen M, Pirttiniemi P (1994). Effect of mechanical forces on chondrocyte maturation and differentiation in the mandibular condyle of the rat. J. Dent. Res..

[215] Sasaguri K, Jiang H, Chen J (1998). The effect of altered functional forces on the expression of bone-matrix proteins in developing mouse mandibular condyle. Arch. Oral. Biol..

[216] Pirttiniemi P, Kantomaa T, Sorsa T (2004). Effect of decreased loading on the metabolic activity of the mandibular condylar cartilage in the rat. Eur. J. Orthod..

[217] Wang X, Mao JJ (2002). Chondrocyte proliferation of the cranial base cartilage upon in vivo mechanical stresses. J. Dent. Res..

[218] Sobue T (2011). Murine TMJ loading causes increased proliferation and chondrocyte maturation. J. Dent. Res..

[219] Tang GH, Rabie ABM, Hägg U (2004). Indian Hedgehog: a mechanotransduction mediator in condylar cartilage. J. Dent. Res..

[220] Shao YY, Wang L, Welter JF, Ballock RT (2012). Primary cilia modulate Ihh signal transduction in response to hydrostatic loading of growth plate chondrocytes. Bone.

[221] Kinumatsu T (2011). TMJ development and growth require primary cilia function. J. Dent. Res..

[222] Woronowicz KC, Gline SE, Herfat ST, Fields AJ, Schneider RA (2018). FGF and TGFβ signaling link form and function during jaw development and evolution. Dev. Biol..

[223] Sprinz R (1965). A note on the mandibular intra-articular disc in the joints of marsupialia and monotremata. Proc. Zool. Soc. Lond..

[224] El Adli JJ, Deméré TA (2015). On the anatomy of the temporomandibular joint and the muscles that act upon it: observations on the gray whale, Eschrichtius robustus. Anat. Rec. Hoboken NJ.

[225] Anthwal N, Tucker AS (2020). The TMJ disc is a common ancestral feature in all mammals, as evidenced by the presence of a rudimentary disc during monotreme development. Front. Cell Dev. Biol..

[226] Marcucio RS, Young NM, Hu D, Hallgrimsson B (2011). Mechanisms that underlie co-variation of the brain and face. Genesis.

[227] Ishii M, Sun J, Ting M-C, Maxson RE (2015). The development of the calvarial bones and sutures and the pathophysiology of craniosynostosis. Curr. Top. Dev. Biol..

[228] Jiang X, Iseki S, Maxson RE, Sucov HM, Morriss-Kay GM (2002). Tissue origins and interactions in the mammalian skull vault. Dev. Biol..

[229] Yoshida T, Vivatbutsiri P, Morriss-Kay G, Saga Y, Iseki S (2008). Cell lineage in mammalian craniofacial mesenchyme. Mech. Dev..

[230] Ting M-C (2009). EphA4 as an effector of Twist1 in the guidance of osteogenic precursor cells during calvarial bone growth and in craniosynostosis. Development.

[231] Deckelbaum RA (2012). Regulation of cranial morphogenesis and cell fate at the neural crest-mesoderm boundary by engrailed 1. Development.

[232] Opperman LA (2000). Cranial sutures as intramembranous bone growth sites. Dev. Dyn..

[233] Lana-Elola E, Rice R, Grigoriadis AE, Rice DPC (2007). Cell fate specification during calvarial bone and suture development. Dev. Biol..

[234] Zhao H (2015). The suture provides a niche for mesenchymal stem cells of craniofacial bones. Nat. Cell Biol..

[235] Maruyama T, Jeong J, Sheu T-J, Hsu W (2016). Stem cells of the suture mesenchyme in craniofacial bone development, repair and regeneration. Nat. Commun..

[236] Wilk K (2017). Postnatal calvarial skeletal stem cells expressing PRX1 reside exclusively in the calvarial sutures and are required for bone regeneration. Stem Cell Rep..

[237] Wu X, Gu Y (2019). Signaling mechanisms underlying genetic pathophysiology of craniosynostosis. Int. J. Biol. Sci..

[238] Agochukwu NB, Solomon BD, Muenke M (2012). Impact of genetics on the diagnosis and clinical management of syndromic craniosynostoses. Childs Nerv. Syst..

[239] Felsenthal N, Zelzer E (2017). Mechanical regulation of musculoskeletal system development. Dev. Camb. Engl..

[240] Opperman LA, Sweeney TM, Redmon J, Persing JA, Ogle RC (1993). Tissue interactions with underlying dura mater inhibit osseous obliteration of developing cranial sutures. Dev. Dyn..

[241] Mabbutt LW, Kokich VG (1979). Calvarial and sutural re-development following craniectomy in the neonatal rabbit. J. Anat..

[242] Moss ML, Young RW (1960). A functional approach to craniology. Am. J. Phys. Anthropol..

[243] Moazen M (2016). Intracranial pressure changes during mouse development. J. Biomech..

[244] Henderson JH, Chang LY, Song HM, Longaker MT, Carter DR (2005). Age-dependent properties and quasi-static strain in the rat sagittal suture. J. Biomech..

[245] Henderson JH, Nacamuli RP, Zhao B, Longaker MT, Carter DR (2005). Age-dependent residual tensile strains are present in the dura mater of rats. J. R. Soc. Interface.

[246] Albright AL, Tyler-Kabara E (2001). Slit-ventricle syndrome secondary to shunt-induced suture ossification. Neurosurgery.

[247] Cohen MM (1993). Sutural biology and the correlates of craniosynostosis. Am. J. Med. Genet..

[248] Hickory WB, Nanda R (1987). Effect of tensile force magnitude on release of cranial suture cells into S phase. Am. J. Orthod. Dentofac. Orthop..

[249] Miyawaki S, Forbes DP (1987). The morphologic and biochemical effects of tensile force application to the interparietal suture of the Sprague-Dawley rat. Am. J. Orthod. Dentofac. Orthop..

[250] Ikegame M (2001). Tensile stress induces bone morphogenetic protein 4 in preosteoblastic and fibroblastic cells, which later differentiate into osteoblasts leading to osteogenesis in the mouse calvariae in organ culture. J. Bone Miner. Res..

[251] Hirukawa K (2005). Effect of tensile force on the expression of IGF-I and IGF-I receptor in the organ-cultured rat cranial suture. Arch. Oral. Biol..

[252] Ogle RC, Tholpady SS, McGlynn KA, Ogle RA (2004). Regulation of cranial suture morphogenesis. Cells Tissues Organs.

[253] Yu JC, Lucas JH, Fryberg K, Borke JL (2001). Extrinsic tension results in FGF-2 release, membrane permeability change, and intracellular Ca++ increase in immature cranial sutures. J. Craniofac. Surg..

[254] Borke JL (2003). Tension-induced reduction in connexin 43 expression in cranial sutures is linked to transcriptional regulation by TBX2. Ann. Plast. Surg..

[255] Lin F-X (2018). Connexin 43 modulates osteogenic differentiation of bone marrow stromal cells through GSK-3beta/Beta-Catenin signaling pathways. Cell. Physiol. Biochem..

[256] Morinobu M (2003). Osteopontin expression in osteoblasts and osteocytes during bone formation under mechanical stress in the calvarial suture in vivo. J. Bone Miner. Res..

[257] Ikegame M, Ejiri S, Okamura H (2019). Expression of non-collagenous bone matrix proteins in osteoblasts stimulated by mechanical stretching in the cranial suture of neonatal mice. J. Histochem. Cytochem..

[258] Shimomura J (2003). Tensile stress induces α-adaptin C production in mouse calvariae in an organ culture: Possible involvement of endocytosis in mechanical stress-stimulated osteoblast differentiation. J. Cell. Physiol..

[259] Choi YH, Choi J-H, Oh J-W, Lee K-Y (2013). Calmodulin-dependent kinase II regulates osteoblast differentiation through regulation of Osterix. Biochem. Biophys. Res. Commun..

[260] Zayzafoon M, Fulzele K, McDonald JM (2005). Calmodulin and calmodulin-dependent kinase IIα regulate osteoblast differentiation by controlling c-fos expression. J. Biol. Chem..

[261] Sun W (2019). The mechanosensitive Piezo1 channel is required for bone formation. ELife.

[262] Persson M (1983). The role of movements in the development of sutural and diarthrodial joints tested by long-term paralysis of chick embryos. J. Anat..

[263] Moss ML (1961). Extrinsic determination of sutural area morphology in the rat calvaria. Acta Anat..

[264] Engström C, Kiliaridis S, Thilander B (1986). The relationship between masticatory function and craniofacial morphology. II. A histological study in the growing rat fed a soft diet. Eur. J. Orthod..

[265] Kaku M (1999). Remodeling of the sagittal suture in osteopetrotic (op/op) mice associated with cranial flat bone growth. J. Craniofac. Genet. Dev. Biol..

[266] Byron CD (2004). Effects of increased muscle mass on mouse sagittal suture morphology and mechanics. Anat. Rec. A. Discov. Mol. Cell. Evol. Biol..

[267] Vecchione L (2007). Craniofacial morphology in myostatin-deficient mice. J. Dent. Res..

[268] Vecchione L (2010). Age related changes in craniofacial morphology in GDF-8 (Myostatin) deficient mice. Anat. Rec. Hoboken NJ.

[269] Kopher RA, Mao JJ (2003). Suture growth modulated by the oscillatory component of micromechanical strain. J. Bone Miner. Res..

[270] Al-Mubarak R, Da Silveira A, Mao JJ (2005). Expression and mechanical modulation of matrix metalloproteinase-1 and -2 genes in facial and cranial sutures. Cell Tissue Res..

[271] Collins JM, Ramamoorthy K, Silveira AD, Patston P, Mao JJ (2005). Expression of matrix metalloproteinase genes in the rat intramembranous bone during postnatal growth and upon mechanical stresses. J. Biomech..

[272] Vij K, Mao JJ (2006). Geometry and cell density of rat craniofacial sutures during early postnatal development and upon in vivo cyclic loading. Bone.

[273] Peptan AI, Lopez A, Kopher RA, Mao JJ (2008). Responses of intramembranous bone and sutures upon in vivo cyclic tensile and compressive loading. Bone.

[274] Soh SH, Rafferty K, Herring S (2018). Cyclic loading effects on craniofacial strain and sutural growth in pigs. Am. J. Orthod. Dentofac. Orthop..

[275] Mosig RA (2007). Loss of MMP-2 disrupts skeletal and craniofacial development, and results in decreased bone mineralization, joint erosion, and defects in osteoblast and osteoclast growth. Hum. Mol. Genet..

[276] Li W (2020). ROCK-TAZ signaling axis regulates mechanical tension-induced osteogenic differentiation of rat cranial sagittal suture mesenchymal stem cells. J. Cell. Physiol..

[277] Barreto S, González-Vázquez A, R. Cameron A, O’Brien FJ, Murray DJ (2017). Identification of stiffness-induced signalling mechanisms in cells from patent and fused sutures associated with craniosynostosis. Sci. Rep..

[278] Cho S, Irianto J, Discher DE (2017). Mechanosensing by the nucleus: from pathways to scaling relationships. J. Cell Biol..

[279] Somech R, Shaklai S, Amariglio N, Rechavi G, Simon AJ (2005). Nuclear envelopathies—raising the nuclear veil. Pediatr. Res..

[280] de Carlos F (2008). Microcephalia with mandibular and dental dysplasia in adult Zmpste24-deficient mice. J. Anat..

[281] Campàs O (2014). Quantifying cell-generated mechanical forces within living embryonic tissues. Nat. Methods.

[282] Zhu M (2020). Spatial mapping of tissue properties in vivo reveals a 3D stiffness gradient in the mouse limb bud. Proc. Natl Acad. Sci. USA.

